# Progress in the Field of Cyclophosphazenes: Preparation, Properties, and Applications

**DOI:** 10.3390/polym16010122

**Published:** 2023-12-29

**Authors:** Omar Dagdag, Hansang Kim

**Affiliations:** Department of Mechanical Engineering, Gachon University, Seongnam 13120, Republic of Korea; omardagdag@gachon.ac.kr

**Keywords:** cyclophosphazenes, preparation, properties, applications, composites, metal complexes, coatings

## Abstract

This review article provides a comprehensive overview of recent advancements in the realm of cyclophosphazenes, encompassing their preparation methodologies, distinctive properties, and diverse applications. The synthesis approaches are explored, highlighting advancements in the preparation of these cyclic compounds. The discussion extends to the distinctive properties exhibited by cyclophosphazenes, including thermal stability characteristics, and other relevant features. Furthermore, we examine the broad spectrum of applications for cyclophosphazenes in various fields, such as coatings, adhesives, composites, extractants, metal complexes, organometallic chemistry, medicine, and inorganic chemistry. This review aims to offer insights into the evolving landscape of cyclophosphazenes and their ever-expanding roles in contemporary scientific and technological arenas. Future possibilities are emphasized, and significant research data shortages are identified.

## 1. Introduction

### 1.1. Recent Advancements in Cyclophosphazenes

Several synthetic approaches have been devised for the preparation of cyclotriphosphazene, involving the chemical modification of cyclotriphosphazenes through reactive functional groups [1]. These strategies yield prepolymers with versatile applications, including but not limited to coatings, adhesives, composites, anticancer medications [2,3], tumor growth inhibitors, flame retardants [4,5,6], biologically active molecules [7,8,9], antimicrobial reagents [10], organic light emitting diodes [10,11,12], catalysis [13,14], fluorescent dyes [15,16,17], polymerization [18,19,20,21,22], gas separation membranes [23], medicinal chemistry [24], lubricant additives [25], and inorganic chemistry [1,26].

The most abundant heterocyclic molecule, hexachlorocyclotriphosphazene (HCCP), has been employed as a precursor in the creation of cyclotriphosphazenes [27], and is a trimer with six active phosphorus and six active chlorine that is easily functionalized by nucleophilic substitution processes involving various nucleophiles, among which are amines, organometallic reagents, aryloxides, alkoxides, alcohols, and carboxyl groups, etc. [1,18,28,29,30].

Cyclotriphosphazenes can have their substituted groups changed to change the physical or chemical characteristics of the final compounds [31]. 

The synthesis of these compounds can be precisely customized by selecting the appropriate nucleophiles. Polymeric structures derived from cyclotriphosphazenes can be categorized into distinct classes, each delineated by the specific nature of their functional groups.

A significant research focus involves synthesizing organophosphazenes that incorporate carboxyl groups [32]. These compounds serve as key components in various applications, including modifiers for dental compositions [33], materials for microencapsulation [34], hydrogels [35], heat-resistant and non-combustible resins [36], hemocompatible films [37], and vaccine adjuvants [38].

### 1.2. Coordination Chemistry of Cyclotriphosphazene Derivatives

Due to their numerous potential uses in the fields of magnetism, optical, porosity, and catalysis, including conductivity, luminescence, non-linear optical materials, and coordination, polymers have received a lot of interest. Many novel structures are now accessible in the literature because of the recent amazing rise in the synthesis of CPs [39,40,41,42,43,44,45]. The metal/ligand stoichiometry, as well as the temperature and concentration, along with the kind of counter ion utilized throughout the reaction process, can all affect how the coordination polymers are made. Additionally, the coordination geometry of the various metal ions used to create these CPs has attracted a lot of study [46,47,48,49,50,51,52,53,54].

Cyclotriphosphazene derivatives are also employed as multi-site coordination ligands to create homo- or heteropoly nuclear complexes with the same or distinct coordination modes [1,55,56].

There are several ways in which cyclotriphosphazenes can interact with the main group and transition metals. (i) These rings’ nitrogen atoms can act as Lewis bases in the presence of the proper Lewis acids, and (ii) covalent or coordinate bonds can be used to include the ring phosphorus atom into a direct metal connection [56]. Additionally, the exo- and endotopic locations of the N-ligands (pyridyloxy, pyrozylyl, etc.) found in cyclotriphosphazene-containing heterocycles can be used as a rough classification [55]. Cyclotriphosphazene-based ligands are employed as fluorescent chemosensors for various metals [7], catalysts [10], and complexing materials due to their structural characteristics [55,57,58]. Additionally, they are used to create coordination polymers [58,59,60]. For instance, the synthesis of coordination polymers (1D, 2D, and 3D) utilizing pyridyloxy cyclotriphosphazene derivatives is possible [1].

The existence of metal complexes with biphenyl groups attached to cyclotriphosphazenes, such as Ru^2+^, Ni^2+^, Cu^2+^, Zn^2+^, Ag^+^, and others, has been reported in the literature [48,61,62]. These complexes often include N-heterocyclic ligands, such as 1,10-phenanthrolino groups, pyridyloxy [16], tetrahydrazone [17], *β*-carboxyethenylphenoxy and p-formylphenoxy groups, diketone groups, amino phosphonate or carboxyl [9], etc.

### 1.3. Cyclotriphosphazene Derivatives as Anticorrosive POLYMERIC Materials

#### 1.3.1. Basics and Socio-Economic Impacts of Corrosion

##### Steel Corrosion

Steel is a simple metal alloy with various mechanical properties [63,64,65]. Steel alloys are continuously being used for different corrosion tests by past and present researchers [66,67,68]. Extensive localized corrosion of steel occurs in high chloride solutions, for example, seawater. This type of corrosion is dangerous environmentally and economically [69,70,71]. For instance, metal pipes and tanks are constructed mainly based on steel, and localized corrosion is a serious problem during crude oil transportation and storage from pipes and tanks [72]. 

#### 1.3.2. Corrosion Protection and Cost

**Figure 1:**
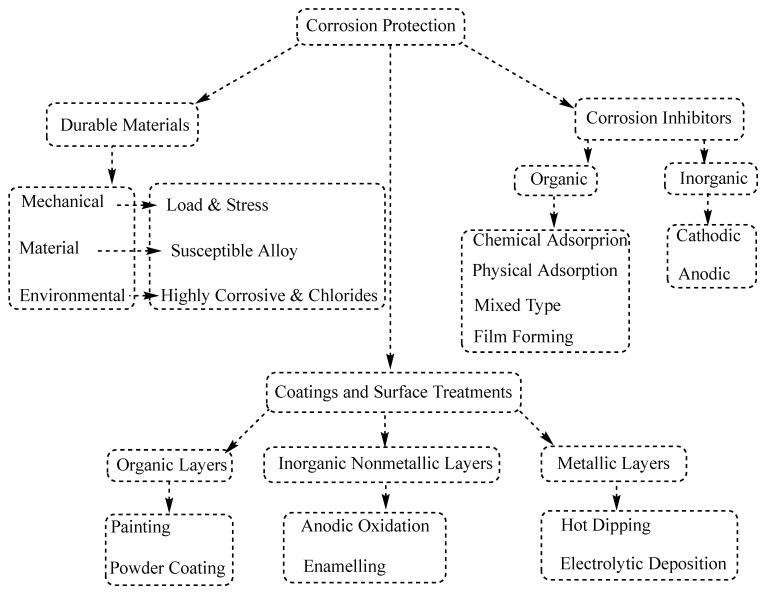
Various ways to protect the metal from corrosion.

There are several methods for protecting metal from the corrosion process; most of them are not sustainable or have a very high production cost. Figure 1 depicts the various methods for protecting the metal from corrosion [73,74,75,76,77,78,79]. As per NACE, USD 2.5 trillion per year worldwide is spent on tackling corrosion problems, which is 3.4% of worldwide GDP [80]. From various countries’ perspectives, South Korea lost USD 1198 billion, USD 1670 billion was lost in India, USD 9330 billion was lost in China, USD 16,950 billion was lost in the EU, USD 3593 billion was lost in Germany, Russia lost USD 2113 billion, and Saudi Arabia lost USD 718 billion due to metal consumption every year. 

##### Corrosion Inhibitors

Corrosion inhibitors containing π-electrons, S, N, O, and P heteroatoms are utilized on metal surfaces for the treatment/protection of metal surfaces from the corrosive environment [81,82,83,84]. This can take place in two ways via adsorption: by physical adsorption or by chemisorption [83]. Physical absorption or physisorption is reversible with a low adsorption enthalpy, typically ranging from 20 to 40 kJ/mol. On the other hand, chemisorption, or chemical adsorption, exhibits irreversibility and involves a higher enthalpy (ΔH) in the range of approximately 80 to 240 kJ/mol. Physisorption is performed at low temperatures and diminishes with increasing temperature and low activation energy (*E*_a_). Chemisorption occurs at room temperature (RT) and increases with the rise in temperature, with high *E*_a_. The corrosion procedure is a characteristic interaction in which the metal is in direct contact with moisture on the corrosion products. In corrosion, hydrogen is formed (cathodic reaction), and dissolution of iron occurs (anodic reaction) [85].

##### Organic Coatings

Thin layers of organic coatings are often placed on the surface of metals to block the entry of corrosive substances. Organic coatings have many benefits, but there is always room for development. For example, they have a limited lifespan and tend to degrade and lose function with time, including their barrier qualities. Some of the elements that govern the durability and longevity of an organic coating include the chemical composition of the coating and the adhesion strength to the metal surface, which is influenced by both the cross-linking density and the type of functional group present on the coating’s surface. 

Epoxy resins based on DGEBA are used in a wide range of industrial applications as composites, matrices, adhesives, coatings, and sealants because they are inexpensive and have great industrial potential. However, because of their brittle behavior and poor anticorrosive qualities, which restrict their usability, it is imperative to enhance these qualities in order to make them appropriate for cutting-edge applications.

The enhancement of anticorrosive characteristics depends heavily on reinforcements and fillers. Additionally, adding compounds that include P, N, and X (halogen), as well as other elements, functions as reinforcement in the polymer matrix, improving the anticorrosive polymers for metals and making them a superior material for coating applications. 

In the current study, the molecule cyclotriphosphazene was chosen as reinforcement for the hybrid matrix because it has high anticorrosive characteristics, an alternate P- and N-connected ring structure, and two reactive substituents contained in the P atom. Consequently, the reason for this review is to research the present status of cyclotriphosphazene derivatives as corrosion inhibitors and anticorrosive polymeric coatings.

## 2. Functional Organic Groups in the Phosphazene Ring: Properties and Applications

### 2.1. Phosphazenes Containing the Carboxyl Group

Phosphazenes and their derivatives containing carboxyl groups are highly intriguing for applications in medicine, electronics, photonics, and the design of versatile high-tech composite materials. They present extensive possibilities for ongoing research and practical utilization [32].

Carboxyl-containing aryloxycyclophosphazenes find application as modifiers for epoxy resins. This is attributed to their potential capacity to serve as resin hardeners, thereby enhancing the performance characteristics of the final materials. However, the compatibility of most carboxyl-containing phosphazenes with resins is hindered by their structural features and the presence of numerous hydrogen bonds.

Hence, Yudaev et al. [18] synthesized a novel carboxyl-containing phosphazene, specifically hexakis-2-(β-carboxyethenylphenoxy)cyclotriphosphazene (2-CEPP), and explored its properties. This investigation is crucial for assessing the compound’s suitability as a modifier for epoxy resins.

**Scheme 1:**
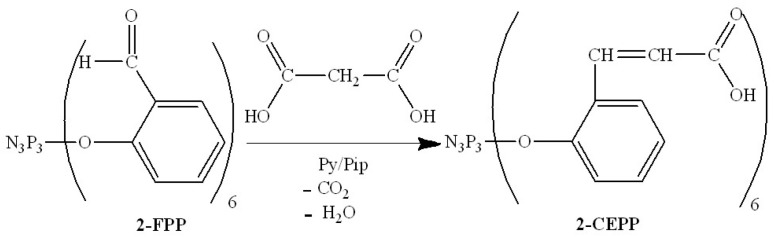
Synthetic pathway for 2-CEPP. Reproduced from reference [18].

2-CEPP was synthesized by reacting hexakis-2-(formylphenoxy) cyclotriphosphazene (2-FPP) with malonic acid. The reaction was conducted by refluxing pyridine using the Knoevenagel–Doebner method [86], as depicted in Scheme 1. The structure of 2-CEPP was verified using ^31^P, ^1^H, and ^13^C NMR spectroscopy, as well as MALDI-TOF mass spectrometry. Through the application of DSC and small-angle X-ray scattering (SAXS), it was discovered that phosphazene exhibits an amorphous state at room temperature. However, upon heating to 185 °C and subsequent rapid cooling, the phosphazene undergoes crystallization. 

**Scheme 2:**
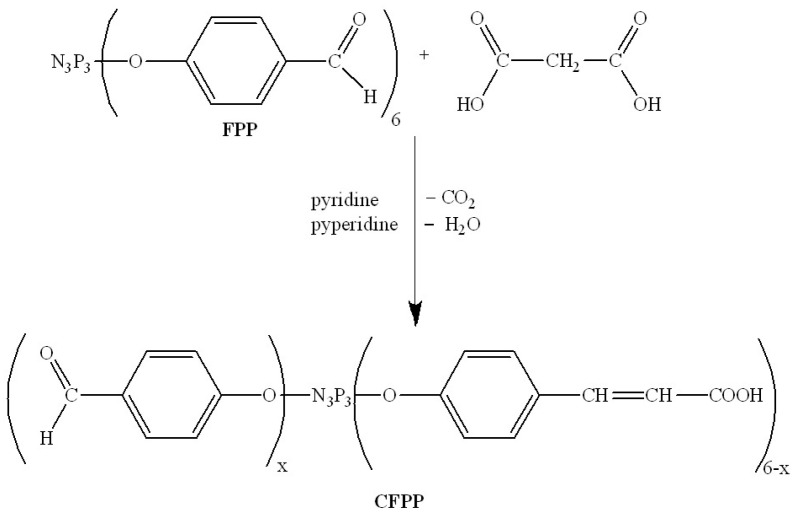
Synthetic scheme for CFPP (x = 1–5). Reproduced from reference [9].

**Scheme 3:**
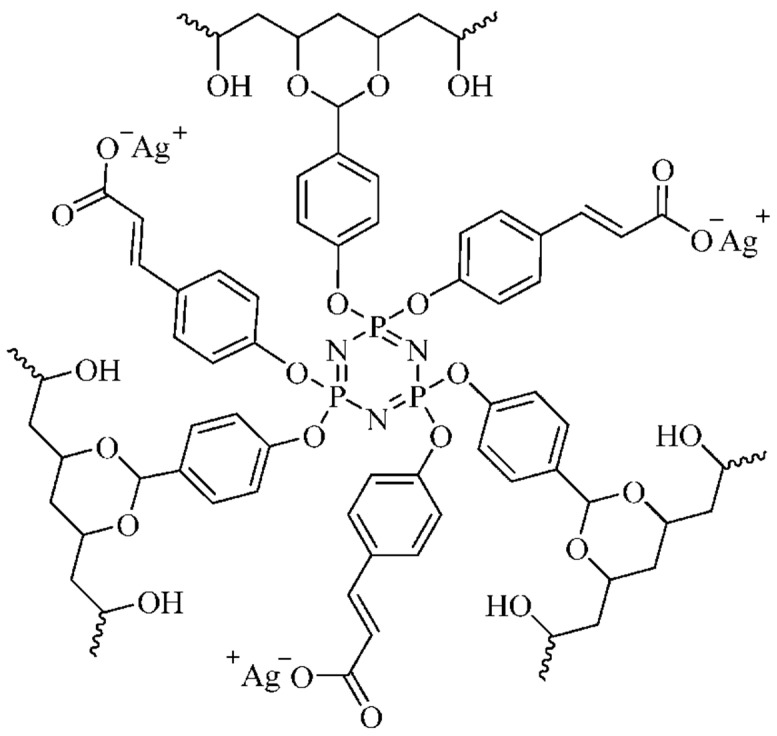
Structure of silver-containing gel. Reproduced from reference [9].

The research group also undertook a project to obtain a wound-healing material containing silver [9]. This material was designed to be non-toxic to the human body, which was achieved by fixing silver ions within the polymer matrix. This novel compound, aryloxycyclotriphosphazene, featuring p-formylphenoxy and β-carboxyethenylphenoxy (CFPP) groups, was employed to inhibit silver aggregation. The numerous aldehyde groups in CFPP facilitated the cross-linking of polyvinyl alcohol (PVA) macromolecules, resulting in gel formation. The β-carboxyethenylphenoxy groups enabled the binding of silver in the form of cinnamates, imparting an antimicrobial effect. The aryloxyclophosphazene CFPP, incorporating β-carboxyethenylphenoxy and p-formylphenoxy groups, was synthesized using the Doebner reaction. This involves the condensation of FPP with malonic acid (MA), a compound featuring an active methylene group, in the presence of a base, as illustrated in Scheme 2. The structure of CFPP was verified using ^31^P, ^1^H, and ^13^C NMR spectroscopy, as well as MALDI-TOF mass spectrometry. The gel demonstrated a maximum water absorption capacity of 272%, achieved after 80 min of testing. The antimicrobial efficacy of the silver-containing gel (Scheme 3) was assessed through the diffusion method. The gel exhibited inhibitory effects on key skin-contact microorganisms, including bacteria such as S. aureus, P. aeruginosa, E. coli, B. subtilis, S. epidermidis, and C. stationis, as well as the fungus C. albicans. Conducting an in vivo study with Pannon rabbits (n = 12) revealed the aerogel’s remarkable wound-healing capabilities. The study observed accelerated epidermal regeneration as early as 3–4 days’ post-treatment. The developed aerogel is suggested for application as an advanced wound dressing, particularly for the efficient healing of acute and open wounds.

### 2.2. Phosphazenes Containing the Amino Group

**Scheme 4:**
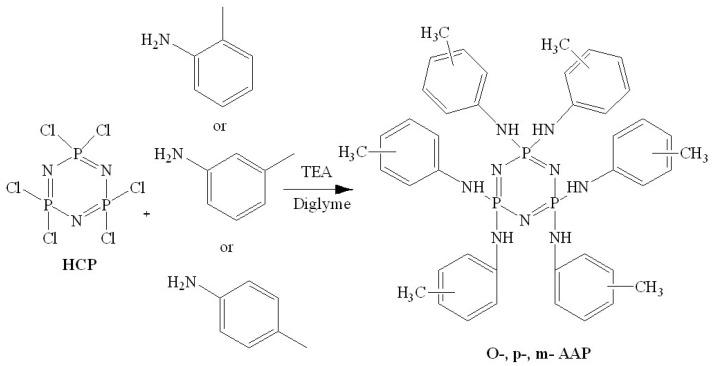
Schematic representation of the synthesis process for AAP. Reproduced from reference [87].

**Scheme 5:**
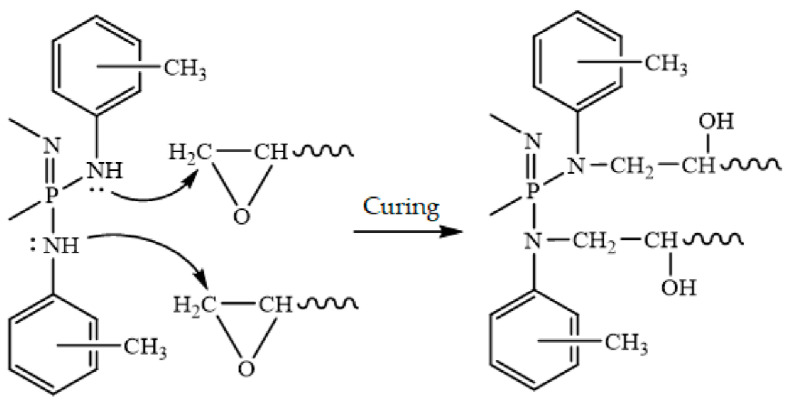
Schematic illustration of the likely interaction between oxirane groups and AAP during the curing process of epoxy resin. Reproduced from reference [87].

Rybyan et al. [87] reported the curing of DER-331 epoxy resin utilizing arylaminocyclotriphosphazenes (AAP) derived from o-, m-, and p-methylanilines. This research highlights the pivotal role of temperature in the rates of chlorine atom replacement in hexa-chlorocyclotriphosphazene (HCP) by the aforementioned methylanilines, elucidating their influence on the specific reaction pathways undertaken. All components of the AAP were synthesized in diglyme following the outlined scheme in Scheme 4. The structural integrity of the synthesized AAP was verified using ^31^P and ^1^H NMR spectroscopy, along with MALDI-TOF mass spectrometry. Utilizing synchronous DSC and TGA, it discerns that the synthesized AAP exhibits a crystalline nature, and its thermal degradation manifests in a distinctive stepped pattern. The process is further characterized by the concurrent removal of three aniline molecules from the AAP entities during thermal destruction. The curing process of epoxy resin DER-331 used the AAP as a curing agent (Scheme 5). It has been determined that owing to steric challenges, o-AAP does not engage with epoxy resin, unlike m- and p-AAP. The gel fraction in the curing resin is quantified, and AAP plays a significant role in the stages of macromolecule formation. As a result, polymers derived from DER-331 and m-, p-AAP exhibit a gel fraction content of up to 97 mass %. These polymers demonstrate glass-transition temperatures of 80 and 85 °C for m- and p-AAP-based compositions, respectively, and exhibit notable fire resistance, conforming to the UL-94 standard with a V-0 classification. 

### 2.3. Phosphazenes Containing the Hydroxyl Group

Cyclotriphosphazenes featuring a hydroxyl (-OH) functional group serve as versatile intermediates for synthesizing a myriad of polymeric compounds. The hydroxyl group can be appropriately modified to yield novel and more advantageous cyclotriphosphazenes.

**Scheme 6:**
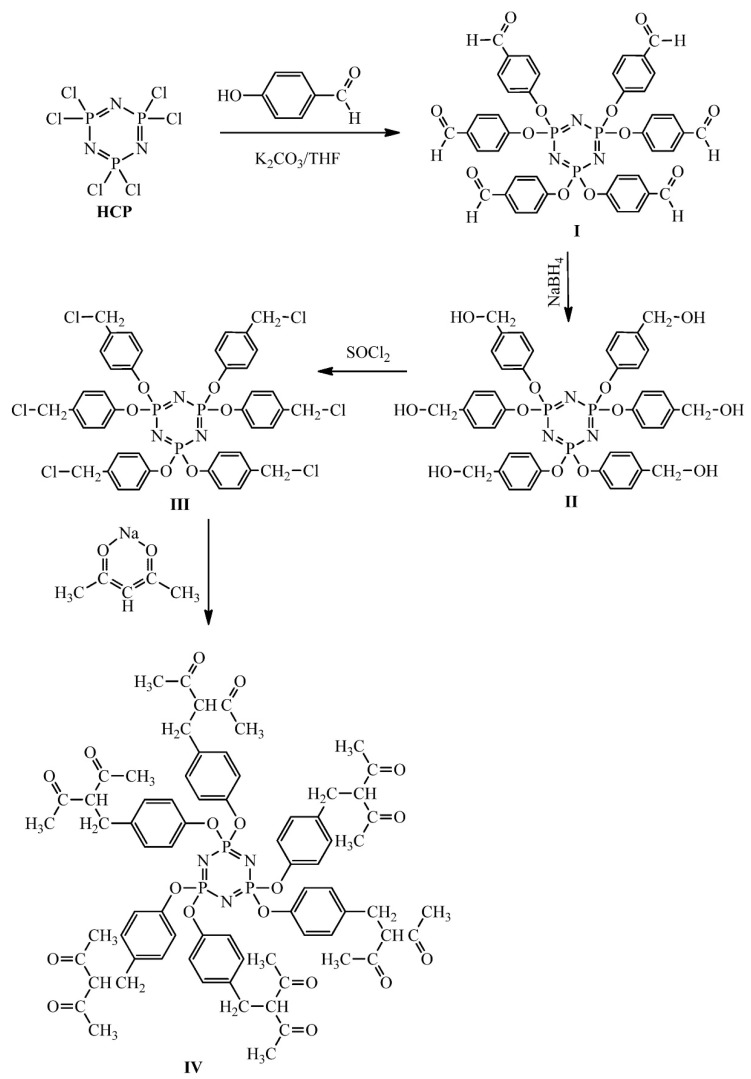
Synthesis of diketophosphazene. Reproduced with permission from reference [20]. Copyright 2019 Elsevier.

**Scheme 7:**
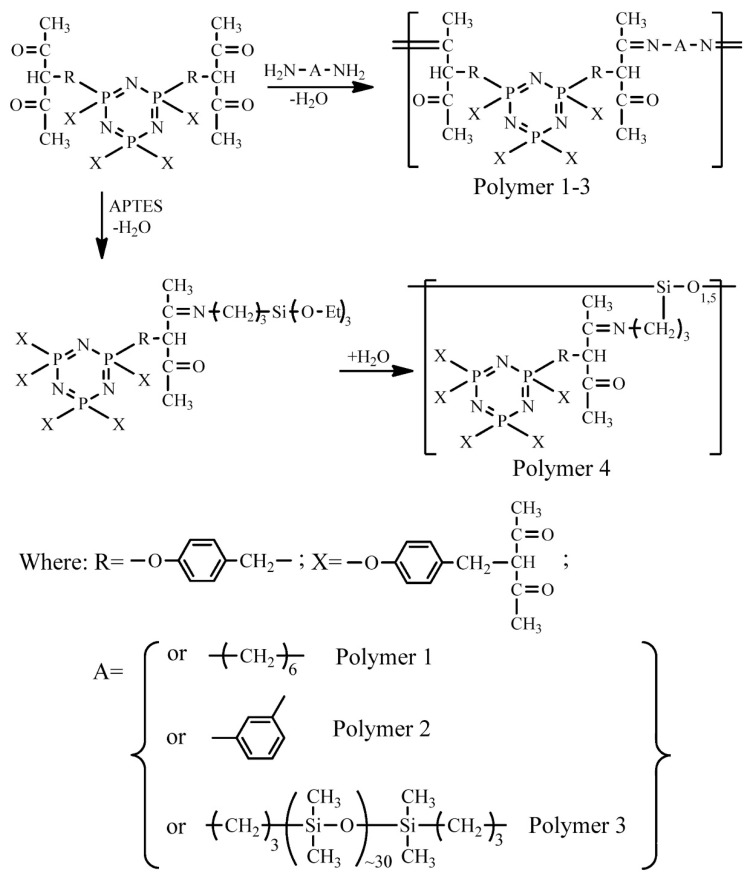
Synthesis of polymers based on the IV and diverse amines. Reproduced with permission from reference [20]. Copyright 2019 Elsevier.

Chistyakov et al. [20] developed films based on β-diketophosphazene and amines to investigate their properties. The aryloxycyclotriphosphazene containing a diketo functional group was synthesized through the reaction of hexakis(4-chloromethylphenoxy) cyclotriphosphazene with sodium acetylacetonate. The synthetic procedure is outlined in Scheme 6. The structure of diketophosphazene was verified using FTIR, ^31^P, ^1^H, and ^13^C NMR spectroscopy and MALDI-TOF mass spectrometry. Polymer films were produced through the interaction of IV with a variety of amines. The formation of the corresponding polymers followed the outlined steps in Scheme 7. It has been established that the polymer derived from IV and (3-aminopropyl) triethoxysilane exhibits superior adhesion to glass, albeit with slightly lower heat resistance compared to other synthesized polymers. Polymer 3, while ranking just below polymer 4 in terms of adhesive characteristics, marginally surpasses it and other polymers in both hydrophobic properties and heat resistance. Consequently, polymer 3 demonstrates the most suitable exploitative properties, making it applicable under relatively extreme conditions. Utilizing this polymer as a foundation, metal-containing films can be generated by forming complexes of diketo groups with metals.

**Scheme 8:**
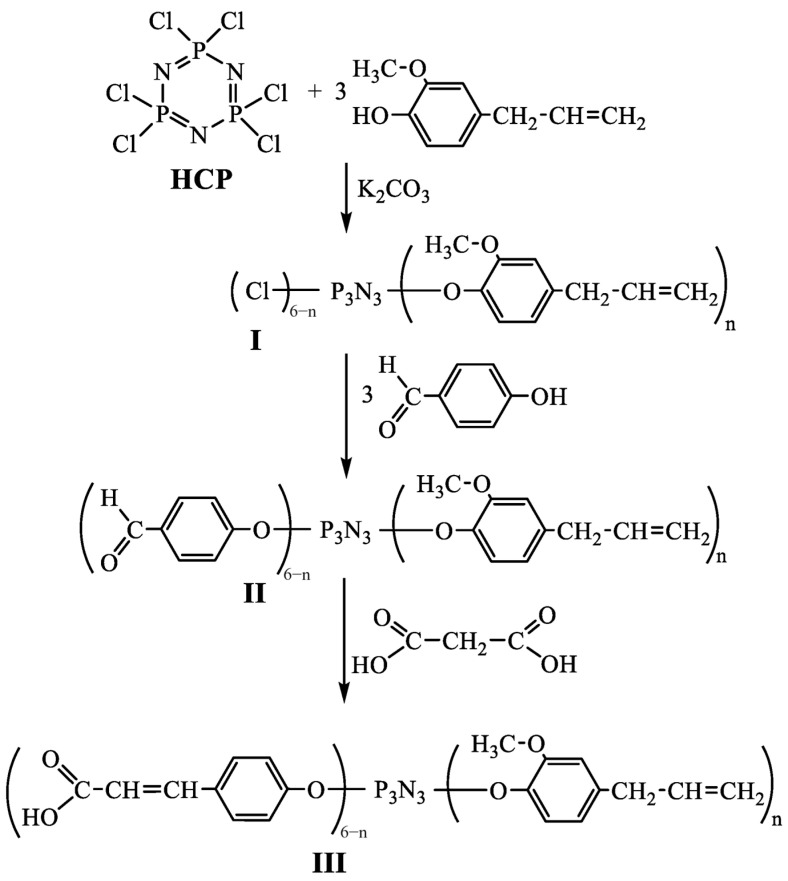
Schematic representation of the preparation process for the cure site monomer used in the dental composition. Reproduced from reference [88].

In a related study by the same research group [88], a novel approach was devised for dental acrylate compositions. This involved incorporating a modifier comprising a blend of cyclotriphosphazenes containing 4-allyl-2-methoxyphenoxy and β-carboxyethenylphenoxy functionalities. Scheme 8 illustrates the synthesis scheme. The synthesized compounds underwent characterization through ^1^H and ^13^C NMR spectroscopy, as well as MALDI-TOF mass spectrometry. Optimal conditions for integrating the modifier with the initial dental mixture, comprising bis-GMA and TGM-3, were determined using the differential scanning calorimetry (DSC) method. The properties of the cured modified compositions were assessed to meet the standards outlined in ISO 4049:2019. These compositions exhibited enhanced adhesion to dental tissues, increased cure depth, and reduced water sorption and water solubility. Moreover, the values of elastic modules, destructive compressive stress, and microhardness demonstrated an upward trend with the escalating content of the modifier in the composition. Based on the results of this study, it can be inferred that the developed dental composition is suitable for practical application as a high-quality and highly adhesive restorative material.

### 2.4. Phosphazenes Containing the Epoxide Group

In recent times, phosphazenes incorporating the epoxide group have emerged as a novel class of compounds with industrial and biological applications. The inclusion of phosphorus-based peripheral functional groups in these compounds enhances their flame-retardant characteristics. The distinctive properties of these phosphorus-based epoxy resins have garnered significant attention among researchers [4,5,89].

**Scheme 9:**
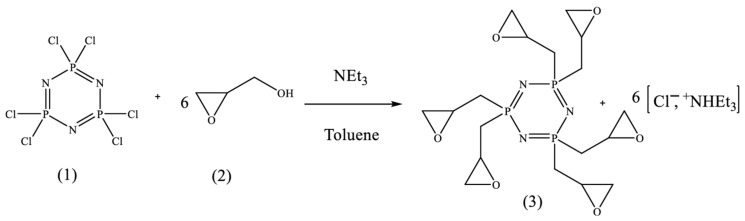
Synthesis pathway for HGCP. Reproduced with permission from reference [91]. Copyright 2009 Elsevier.

In our previous studies [90,91], our group devised a method for synthesizing hexaglycidyl cyclotriphosphazene (HGCP) by condensing glycidol with hexachlorocyclotriphosphazene. This process involved a one-step reaction, encompassing the generation of a nucleophilic group through the deprotonation of the glycidol hydroxyl group, followed by nucleophilic substitution. This substitution led to the displacement of chloride from hexachlorocyclotriphosphazene (HCCP), resulting in the formation of the condensation product and a salt. The synthesis details are succinctly outlined in Scheme 9. An advantageous aspect of this method lies in the versatility of the developed compounds, which can be utilized either as a precursor for creating composite materials or as a reactive flame-retardant additive. The HGCP was subsequently cross-linked with the methylene dianiline (MDA) hardener in our experimental setup. The hexaglycidyl cyclotriphosphazene (HGCP) with the MDA hardener yields a film that is tough, hard, insoluble, and infusible. This HGCP variant exhibits notable thermal stability at elevated temperatures and displays excellent flame-retardant properties. According to the results of differential scanning calorimetry (DSC) analysis, the glass transition temperature (*T*_g_) increased from 77 °C (for HGCP-MDA) to 93 °C for the DGEBA-20%HGCP-MDA thermoset.

An alternative method for the synthesis of epoxy phosphazenes involves the epoxidation of phosphazenes containing double bonds using peracids [21]. 

**Scheme 10:**
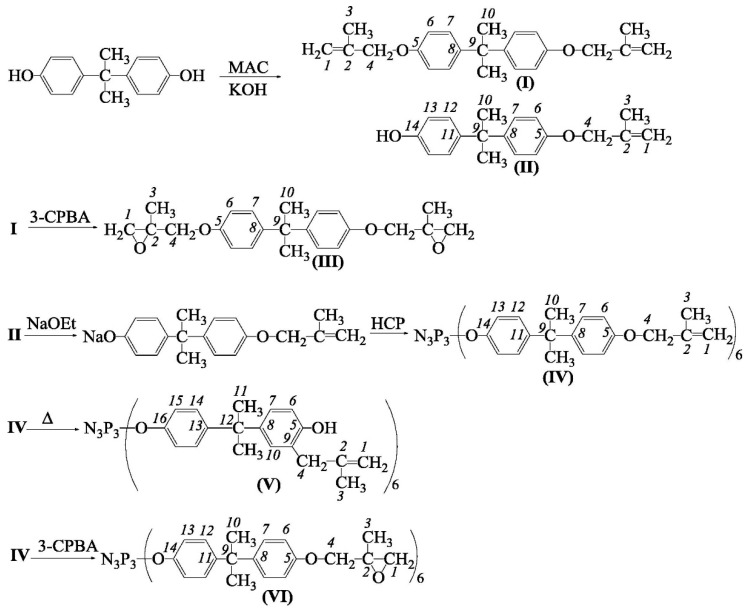
Synthesis of epoxy compound VI. Reproduced with permission from reference [21]. Copyright 2019 Elsevier.

Chistyakov et al. [21] synthesized another new epoxy compound based on 4-(2-(4-((β-Methallyl)oxy)phenyl)propan-2-yl)phenol, which was prepared via the reaction of methalyl chloride with bisphenol A and then was employed in the synthesis of hexakis-4-(2-(4-((β-methallyl)oxy)phenyl)propan-2-yl)phenoxycyclotriphosphazene. The synthetic procedure is outlined in Scheme 10. It was discovered that the latter undergoes the Claisen rearrangement and is susceptible to epoxidation by 3-chloroperbenzoic acid. The resulting epoxide was subsequently cured through treatment with isophorone diamine. Additionally, the decomposition and glass transition temperatures of the cured resin were determined using DSC and TGA methods, yielding values of 275 and 130 °C, respectively. The resin, derived from the synthesized epoxide, exhibited noteworthy thermal characteristics, rendering it suitable for incorporation into adhesive formulations.

### 2.5. Phosphazenes Containing Extractant

**Scheme 11:**
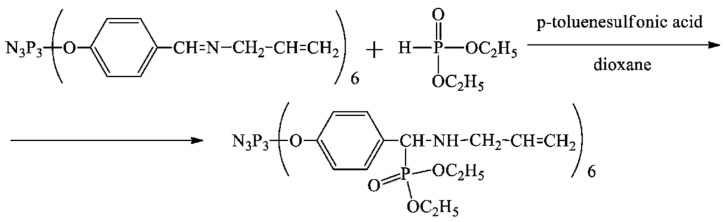
Synthetic scheme for extractant preparation. Reproduced from reference [92].

**Scheme 12:**
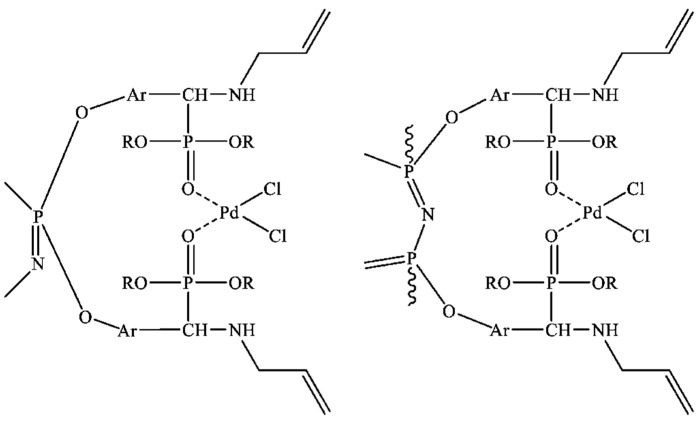
Chelate complexes of palladium(II) with the extractant, featuring intrasphere coordination of palladium (R = C_2_H_5_, Ar = p-C_6_H_4_). Reproduced from reference [92].

Yudaev et al. [92] developed a highly efficient and environmentally friendly method for extracting palladium from hydrochloric acid media. The extractant was synthesized through the Pudovik reaction involving hexakis-[4-{(N-allylimino)methyl}-phenoxy]-cyclotriphosphazene (APP) and diethyl phosphite in dioxane, as illustrated in Scheme 11. The extractant underwent characterization through ^1^H, ^13^C, and ^31^P NMR spectroscopy, as well as MALDI TOF mass spectrometry, while the palladium complex derived from it was characterized using IR spectroscopy. The sorption efficiency of palladium(II) using the developed sorbent can attain 71% in a single cycle. Following treatment of the spent sorbent with 5 M hydrochloric acid, complete extraction of palladium from the sorbent is achieved. The results indicate the possibility of forming structurally diverse chelate complexes, as illustrated in Scheme 12. This novel sorbent is suggested for the extraction of palladium from hydrochloric acid media, particularly those obtained through the leaching of electronic waste.

### 2.6. Phosphazenes Containing Metal Complexes

A significant group of inorganic heterocyclic rings with the repetition unit [-N = PX_2_-] are known as cyclophosphazenes. The most prevalent constituents of these heterocyclic substances are N_3_P_3_C_6_ and N_4_P_4_C_8_. Because of the numerous nucleophilic substitution processes that occur in trimer and tetramer derivatives, there is much research on them. The creation of novel compounds with various features, such as liquid crystal, physiologically active, and photophysically active molecules, begins with cyclic compounds [93,94]. By substituting the Cl atoms with nitrogen and oxygen atoms on the P atoms, cyclotriphosphazenes have also been gaining interest as ligands in coordination chemistry. In recent years, starting materials for the creation of multi-site polydentate ligands have been N_3_P_3_C_l6_ derivatives in particular [48,60,95]. 

One notable area of investigation might be the creation and production of metal complexes that contain cyclotriphosphazene ligands.

**Scheme 13:**
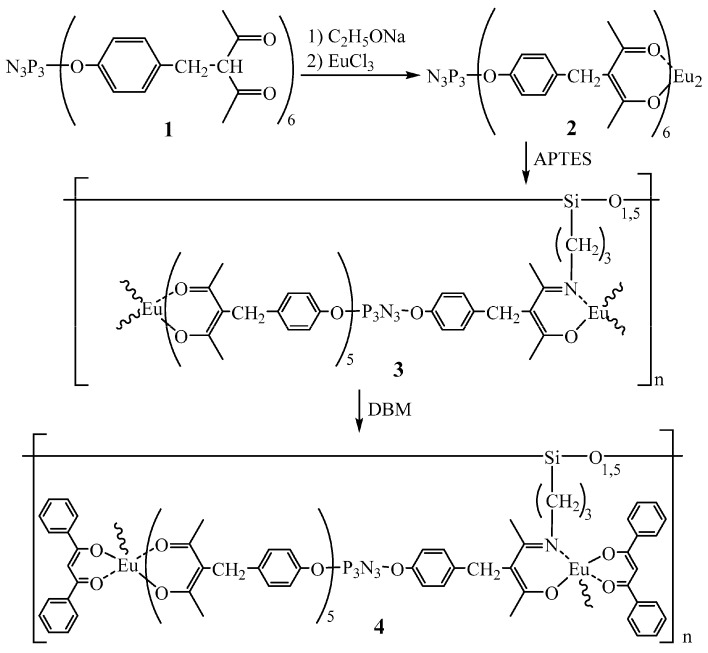
Schematic representation of the synthesis of compounds **1**–**4**. Reproduced from reference [96].

Maslennikova et al. [96] developed a polymer through the reaction of β-diketophosphazene with europium(III) salt. The synthesized metal complex was further structured by incorporating (3-aminopropyl)triethoxysilane and treated with dibenzoylmethane to facilitate additional coordination of europium atoms. The synthesis of the europium-containing polymer (compound **4**) was carried out through multiple steps following the scheme depicted in Scheme 13. The research yielded a polymer suitable for utilization in the form of films and coatings, serving as a source of monochromatic red light with a wavelength of 615 nm. The polymer exhibits thermal stability up to 300 °C, the corresponding coating demonstrates a contact angle of 101°, and its adhesive strength to non-finished glass, according to ISO 2409:2013, is rated at 1 point.

**Scheme 14:**
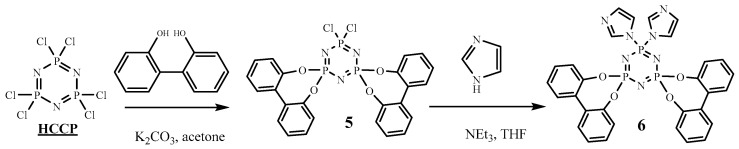
Synthesis of compound **6**. Reproduced with permission from reference [97]. Copyright 2020 Elsevier.

İbişoğlu et al. [97] reported the synthesis of [N_3_P_3_(biph)_2_(Im)_2_] (**6**) obtained with ImH as monodentate base and compound (**5**) using NEt_3_ in boiling THF [97]. The HCCP reacted with the diol 2,2′-HOC_6_H_4_C_6_H_4_OH in the presence of K_2_CO_3_ in acetone to produce the compound (**5**) ({N_3_P_3_(biph)_2_Cl_2_}c) [98]. The synthetic procedure for the (**6**) ligand is presented in Scheme 14.

**Scheme 15:**
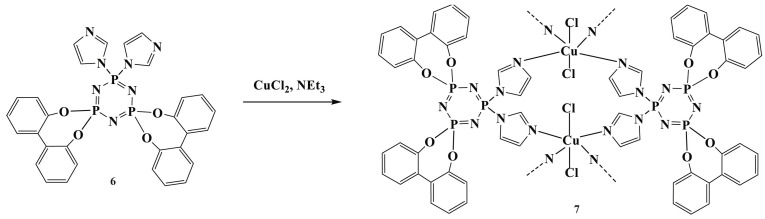
Synthesis of 1D Cu^2+^ coordination polymer (**7**). Reproduced with permission from reference [97]. Copyright 2020 Elsevier.

**Figure 2:**
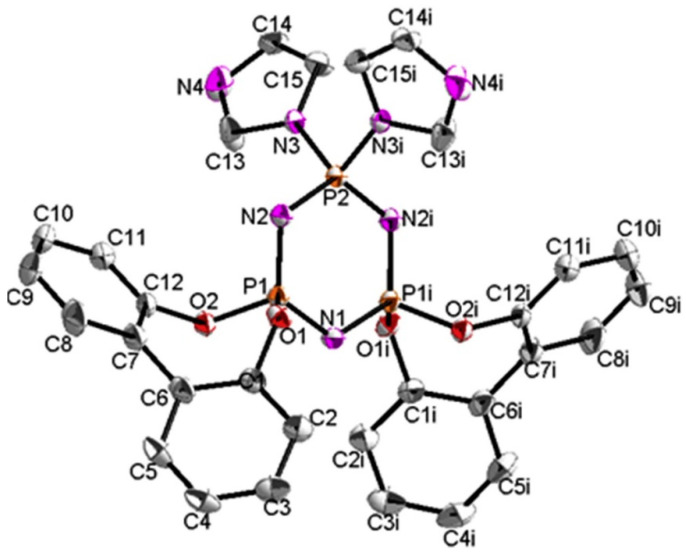
The crystal structure of **8**. Reproduced with permission from reference [97]. Copyright 2020 Elsevier.

**Figure 3:**
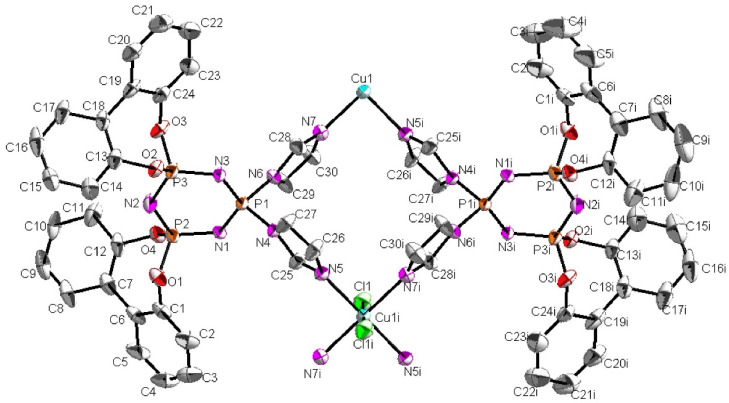
The crystal structure of **9**. Reproduced with permission from reference [97]. Copyright 2020 Elsevier.

Bişolu et al. [18] described the synthesis of the 1D Cu^2+^ coordination polymer (**7**) (Scheme 15) and the [L2(CuCl_2_)]n complex imidazole along with biphenyl-appended cyclotriphosphazene (**6**) (Scheme 15). Spectroscopic investigations (IR, ^31^P, and ^1^H NMR), MALDI-MS, and elemental analysis were used to identify the structure of **7**. Single-crystal X-ray (SCXRD) was used to establish the crystal structures of **8** and **9** (Figure 2 and Figure 3). The structure of the six-coordinate Cu^2+^ complex (**9**) was produced by two cyclotriphosphazene rings connected by di-N-Cu-N bridges, according to SCXRD data. The Cu^2+^ complex of cyclotriphosphazene displayed octahedral geometry.

**Figure 4:**
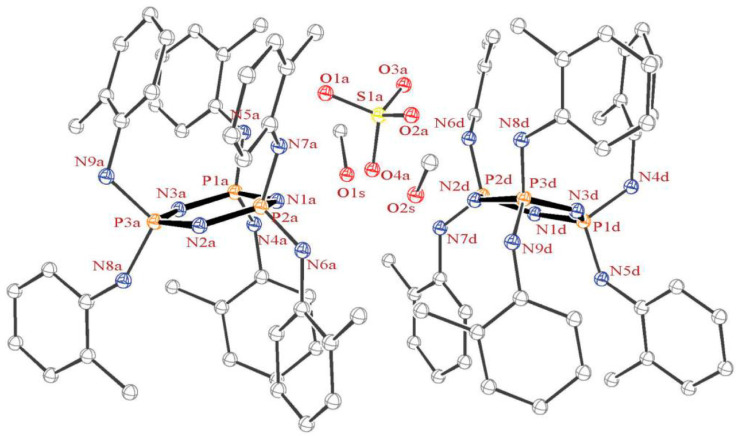
The crystal structure of **10**. Reproduced with permission from reference [99]. Copyright 2020 Elsevier.

**Figure 5:**
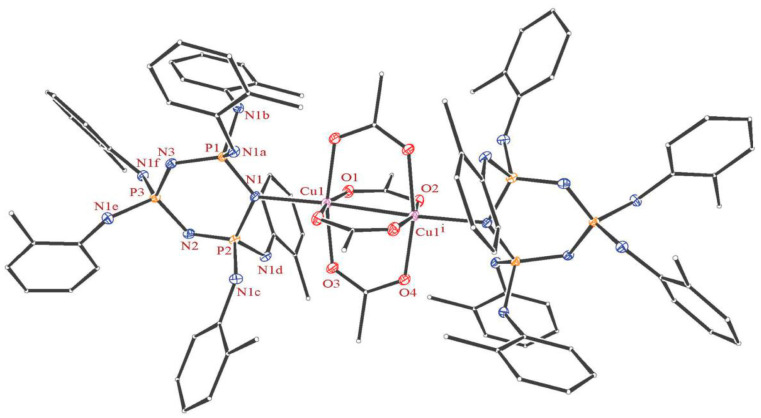
The crystal structure of **11**. Reproduced with permission from reference [99]. Copyright 2020 Elsevier.

Hakimi et al. [99] reported the coordinating polymeric compounds, namely [HMPAP]_2_[SO_4_]·2CH_3_OH (**10**) and [Cu(μ-OAc)_2_(MPAP)]_2_ (**11**), have been synthesized, and the integrity of the compounds was determined using elemental analysis, Raman spectroscopy, and FTIR. The crystal structures of **10** and **11** were defined by SCXRD (Figure 4 and Figure 5).

In the crystal structure of **10**, one nitrogen atom of the cyclotriphosphazene ring has been protonated. The copper complex (**11**) is binuclear formed by four bridging acetato ligands containing one Cu–Cu bond in the center of the complex.

**Scheme 16:**
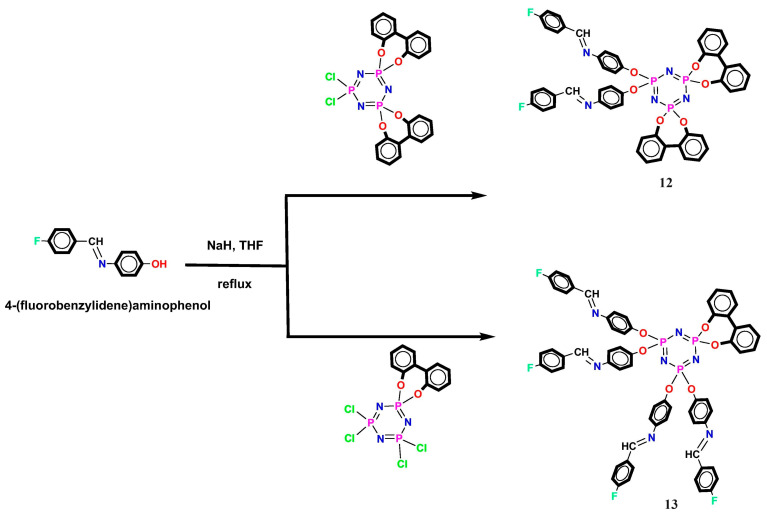
The combination of **12** and **13**. Reproduced with permission from reference [100]. Copyright 2020 Elsevier.

**Figure 6:**
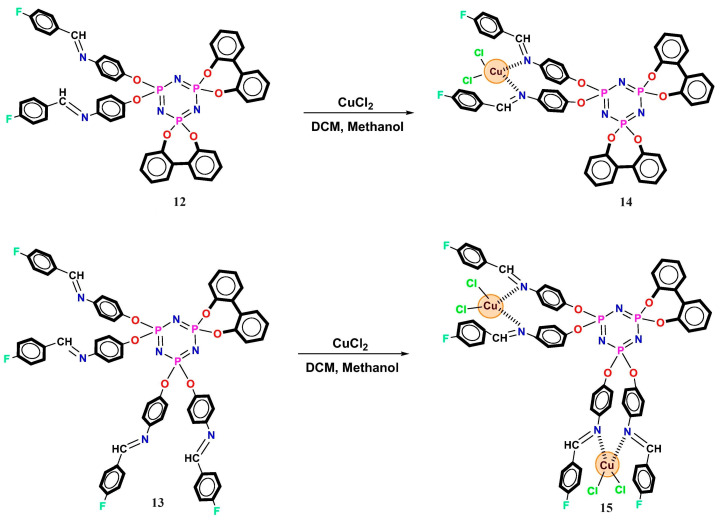
The way that **14** and **15** coordinate. Reproduced with permission from reference [100]. Copyright 2020 Elsevier.

**Figure 7:**
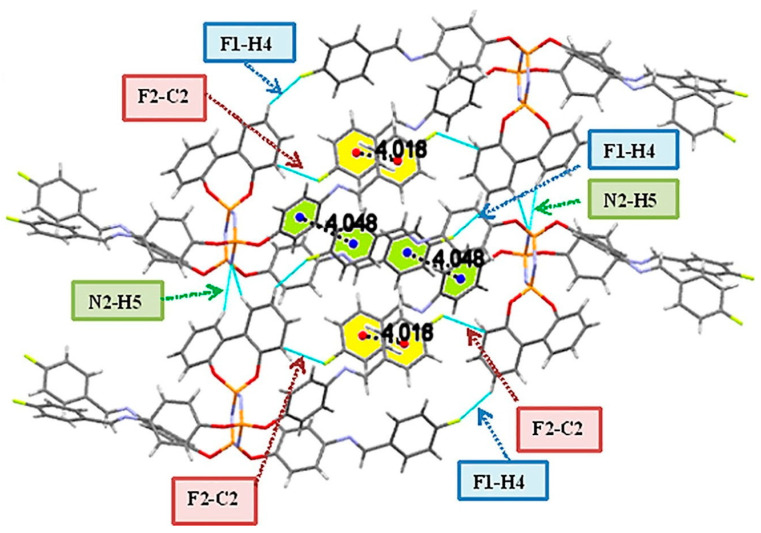
Perspective views of some the intermolecular interactions in **13**. Reproduced with permission from reference [100]. Copyright 2020 Elsevier.

**Figure 8:**
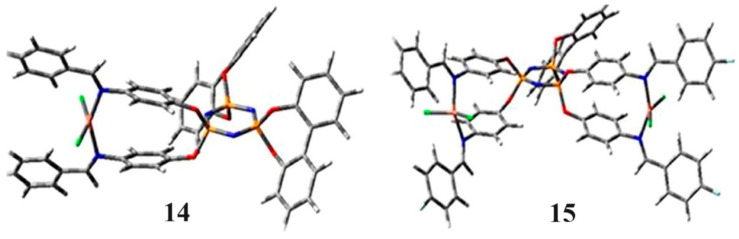
Optimized geometries of **14** and **15** with the basis set B3LYP/6-31G (d, p). Reproduced with permission from reference [100]. Copyright 2020 Elsevier.

Additionally, **12** and **13** were synthesized, according to Doğan et al. [100]. Scheme 16 presents the **12** and **13** synthesis pathways. By using elemental analysis, ^1^H, ^31^P NMR, and FT-IR spectroscopy, the structures of both **12** and **13** were identified. The complexation processes of both **12** and **13** with CuCl_2_ salts were used to create **14** and **15** (Figure 6). A single molecule is present in the asymmetric unit of **13**, and the crystallographic investigation reveals that **13** crystallized in the monoclinic space group *C1 2/c1* (Figure 7). DFT computations provided support for the coordination mode of the planned Cu^2+^ complexes **14** and **15**. Figure 8 illustrates the optimized geometry of both **14** and **15**. The investigations showed that the metal ion coordinates to two imine groups of geminal Schiff bases in the ƞ^2^*-geminal-N*_2_ coordination mode in the two new mononuclear and dinuclear Cu^2+^ complexes (**14** and **15**).

**Scheme 17:**
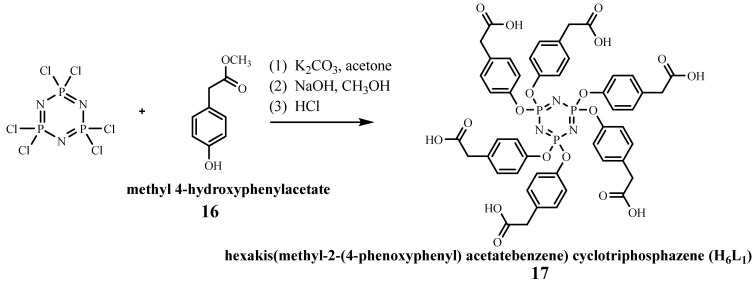
Synthesis of compound **17**. Reproduced with permission from reference [101]. Copyright 2023 Elsevier.

Erkovan et al. [101] synthesized ligand H_6_L_1_ (**17**) (illustrated in Scheme 17) through the following steps: Firstly, the HCCP reacted with the methyl 4-hydroxyphenylacetate (**16**) in the presence of K_2_CO_3_ in acetone. Secondly, the NaOH was dissolved in methanol. The reaction mixture was then supplemented with N_3_P_3_(OC_6_H_4_CH_3_COOCH_3_). The resulting mixture was heated to 80 degrees Celsius and stirred overnight at that temperature. It was then filtered, and the pH was adjusted to **2**–**3** using diluted HCl.

**Figure 9:**
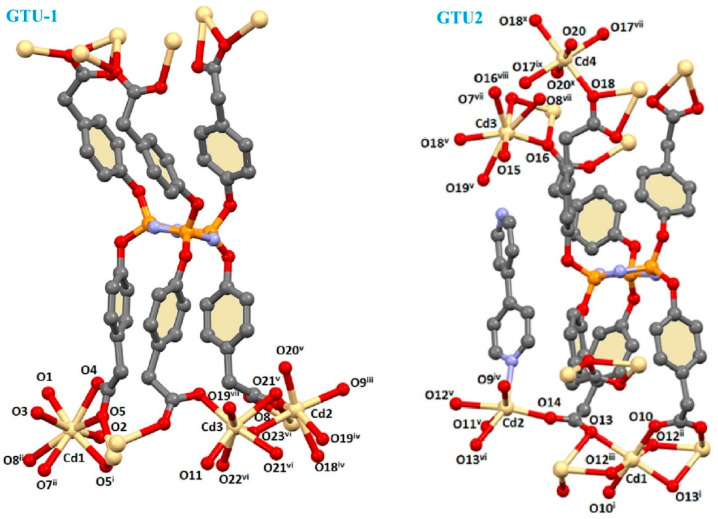
Environment for Cd(II) coordination in GTU-1 (**18**) and GTU-2 (**19**). Reproduced with permission from reference [101]. Copyright 2023 Elsevier.

H_6_L_1_ was used in the synthesis along with the structural elucidation of a polymeric as well as two 3D Cd^2+^ CPs by Erkovan et al. [31]. These are the coordinating polymeric compounds: GTU-1 (**18**) and GTU-2 (**19**). FTIR spectroscopy, powder X-ray diffraction, and SCXRD analyses were used to characterize Cd(II) coordination polymers. GTU-1 (**18**) crystallizes in the monoclinic space group C2/c, according to the crystallographic investigation, while GTU-2 (**19**) crystallizes in the triclinic P-1 space group, displaying a 3D coordination polymer (CP) (Figure 9). While GTU-2 (**19**) is a 3D CP made from 1D inorganic building unit (IBU) joined by deprotonated H_6_L_1_ (**17**) and carefully selected ancillary linker 4,4-bipyridine, GTU-1 (**18**) is a 3D pillared-layered CP structure made up of 2D Cd-carboxylate (Cd(COO)_n_)-based IBU. Both complexes completely deprotonate the adaptable multi-site cyclotriphosphazene bridging ligand (H_6_L_1_) (**17**).

**Figure 10:**
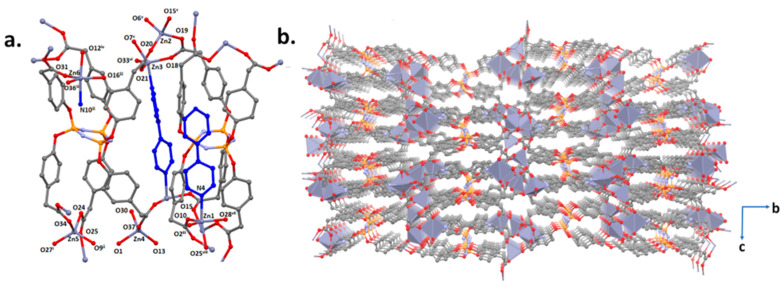
GTU-3’s (**20**) crystal structure as shown (**a**), and a view of its three-dimensional coordination (**b**). Reproduced from reference [102].

Using the same ligand H_6_L_1_ (**17**), the 3D Zn^2+^ CP GTU-3 (**19**) was created. Scheme 17 displays the ligand’s synthetic strategy. FTIR, powder, and SCXRD analyses, were used to characterize the Zn^2+^coordination polymer. Two completely deprotonated cyclophosphazene-based ligands, six independently crystallographic Zn^2+^ ions, and two 4,4′-bipyridines make up GTU-3 (**20**) (Figure 10a). The monoclinic crystal structure of GTU-3 (**20**) crystallizes with the *P*2_1_/n space group. Figure 10b depicts a 3D coordination polymer by an a-axis. 

**Scheme 18:**
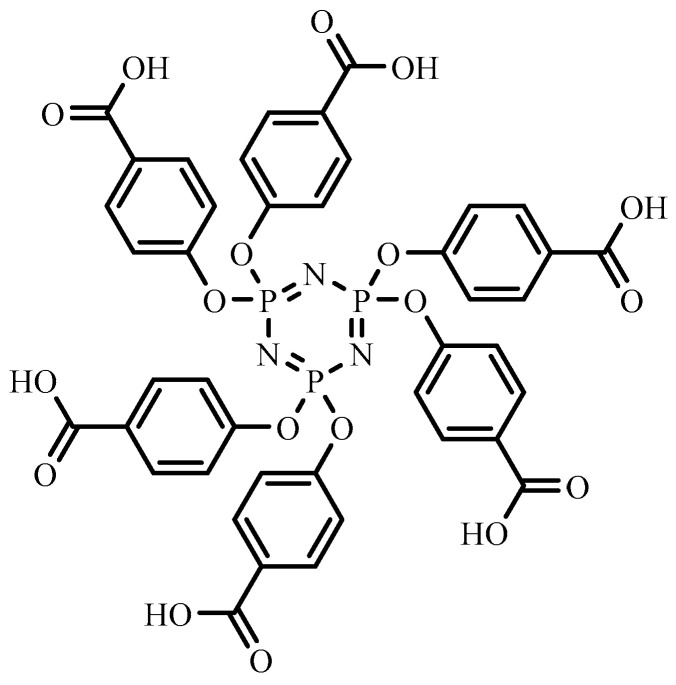
H_6_L_1_ ligand structure (**21**). Reproduced with permission from reference [103]. Copyright 2015 American Chemical Society.

**Figure 11:**
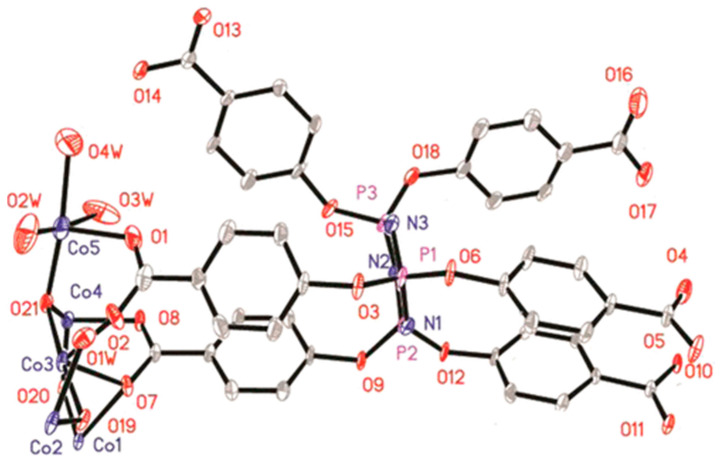
Perspective of the one-dimensional asymmetric unit of compound **22**. Reproduced with permission from reference [103]. Copyright 2015 American Chemical Society.

**Figure 12:**
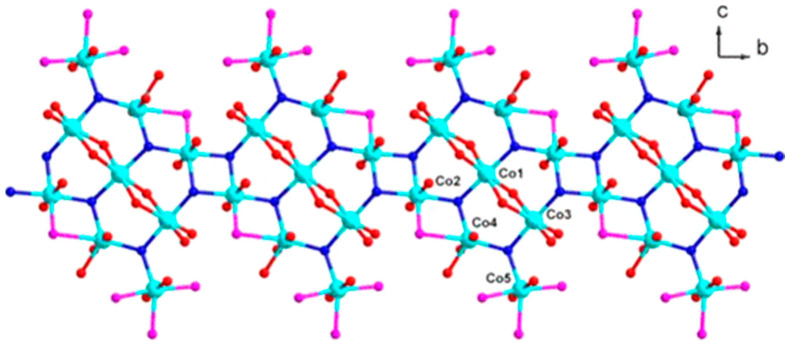
A perspective image of the 1D cobalt-hydroxyl chain. Reproduced with permission from reference [103]. Copyright 2015 American Chemical Society.

**Figure 13:**
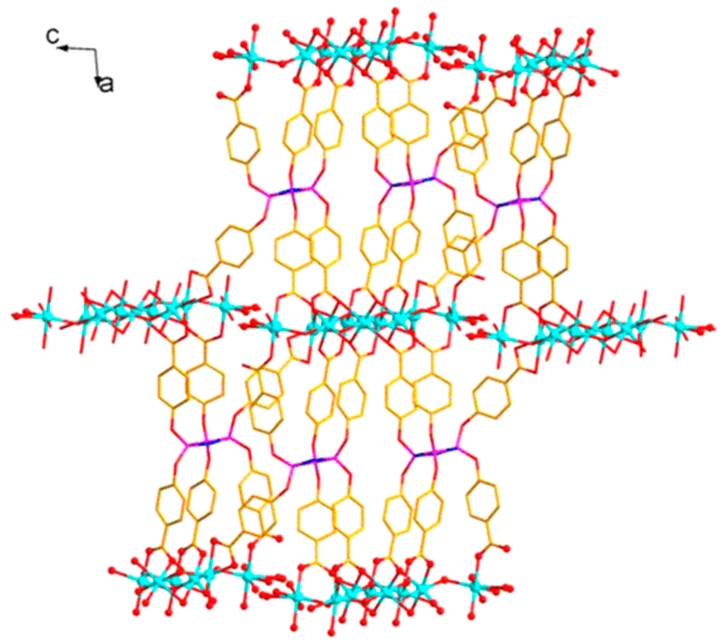
A perspective of **22**’s 3D structure. Reproduced with permission from reference [103]. Copyright 2015 American Chemical Society.

H_6_L_1_ ligand (**21**) (Scheme 18) has been successfully used in another study with Li et al. [39] to build the novel 3D magnetic network [Co_9_(OH)_6_(C_42_H_24_O_18_P_3_N_3_)_2_(H_2_O)_8_] (**22**). In the asymmetric unit of compound **22**, which crystallizes in the monoclinic space group *P*2_1_/*c*, there are three independent -OH ions, four coordinated aqua molecules, three full hexacarboxylate ligands (Co2-Co5), four complete Co ions (Co2-Co5), as well as a half-occupied Co ion (Co1) (Figure 11). The almost two-dimensional cobalt layer is made up of 1D Co-OH chains formed by planar nonanuclear clusters that are positioned in the b–c plane (Figure 12). Figure 13 displays the 3D structure of 1 from a perspective.

**Scheme 19:**
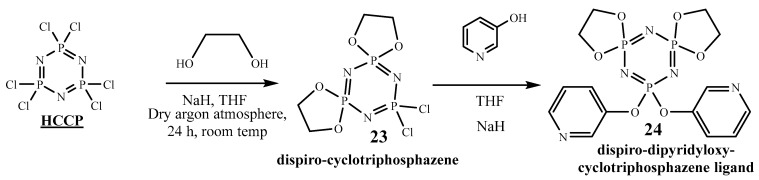
Synthesis of compound **24**. Reproduced with permission from reference [104]. Copyright 2017 Elsevier.

Davarcı et al. [104] synthesized a new ligand (**24**) by an SN reaction involving dispiro-cyclotriphosphazene compound (**23**) and 3-hydroxypyridine in the presence of NaH as the base (Scheme 19). 

In order to create a novel Ag(I) complex, Davarc et al. [29] created the ligand dispiro-dipyridyloxy-cyclotriphosphazene (L) (**24**). 

**Figure 14:**
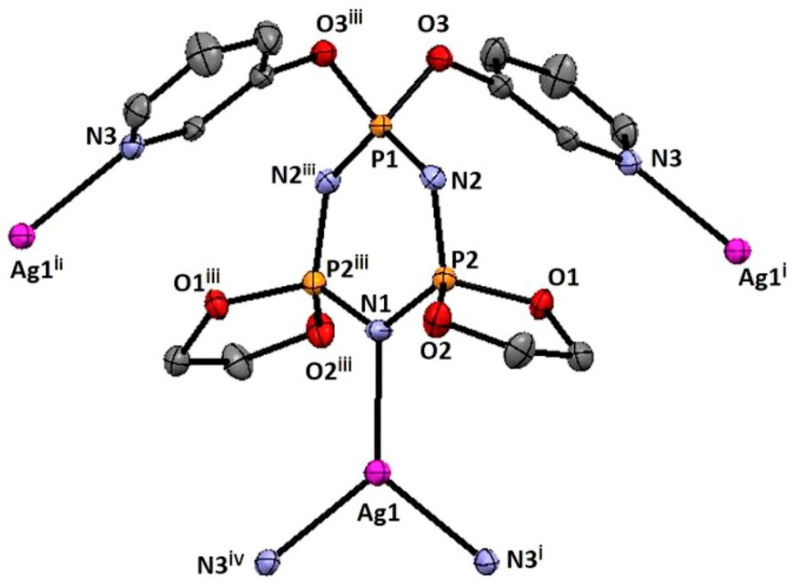
Spirocyclic phosphazene coordination polymer’s crystal structure (**25**). Reproduced with permission from reference [104]. Copyright 2017 Elsevier.

**Figure 15:**
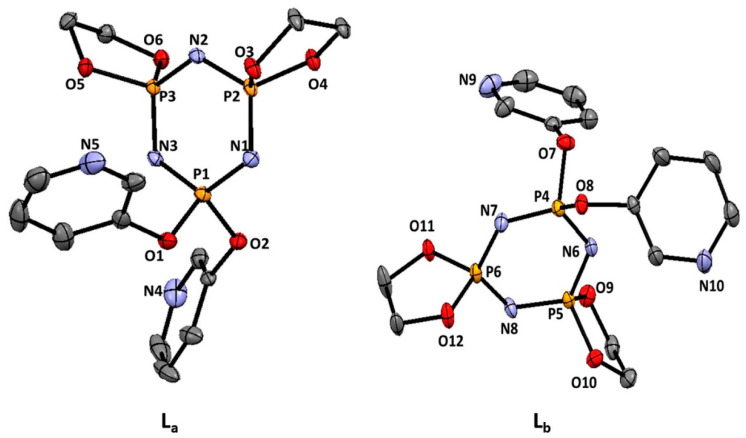
The ligand’s crystal structure is (L_a_−L_b_). Reproduced with permission from reference [104]. Copyright 2017 Elsevier.

Utilizing AgPF_6_ salt, ligand (**24**) was converted into Ag CP (**25**) (Figure 14), which adopts a κ3N coordination mode and possesses a 1D structure. Spectroscopic investigations (IR, ^31^P, and ^1^H NMR), mass spectrometry (MS), and elemental analysis were used by the authors to discover the molecular formulas of synthetic substances. Additionally, XRD’s structural determination of L along with compound **25**. Dichloromethane/hexane was used to produce colorless crystals of L. In accordance with crystallographic studies, L crystallized in the monoclinic space group P21/n. Figure 15 depicts both L_a_ and L_b_, two distinct molecules with differing symmetries that make up an asymmetric unit cell. A 1D polymeric structure was produced by L’s κ3N coordination mode involving Ag^+^ ions in complex **26**. When compared to the free ligand (L), complex (**25**)’s P-N stretching frequencies displayed some variations, according to IR spectra. TGA study of L and CP 1 revealed that L has greater thermal stability than complex (**25**).

**Figure 16:**
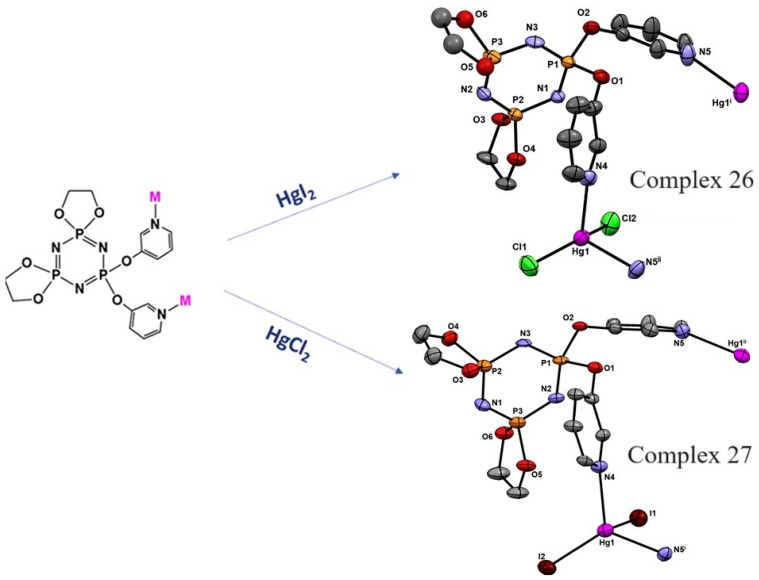
Complexes **26** and **27**’s crystal structures. Reproduced with permission from reference [28]. Copyright 2019 Elsevier.

Another new two Hg^2+^ cyclotriphosphazene CPs were synthesized with the same ligand L [104], namely {[Hg(L)(I)_2_]}_n_ (Complex **26**) and {[Hg(L)(Cl)_2_]}_n_.(can)_0,341_ (Complex **27**), and their crystal structures are shown in Figure 16. The XRD analysis revealed that **26** and **27** crystallize in the orthorhombic (space group *Pccn*) and the monoclinic crystal system (space group *P*2_1_/c) [28]. The central Hg^2+^ ion in **26** and **27** has a distorted tetrahedral coordination geometry.

**Scheme 20:**
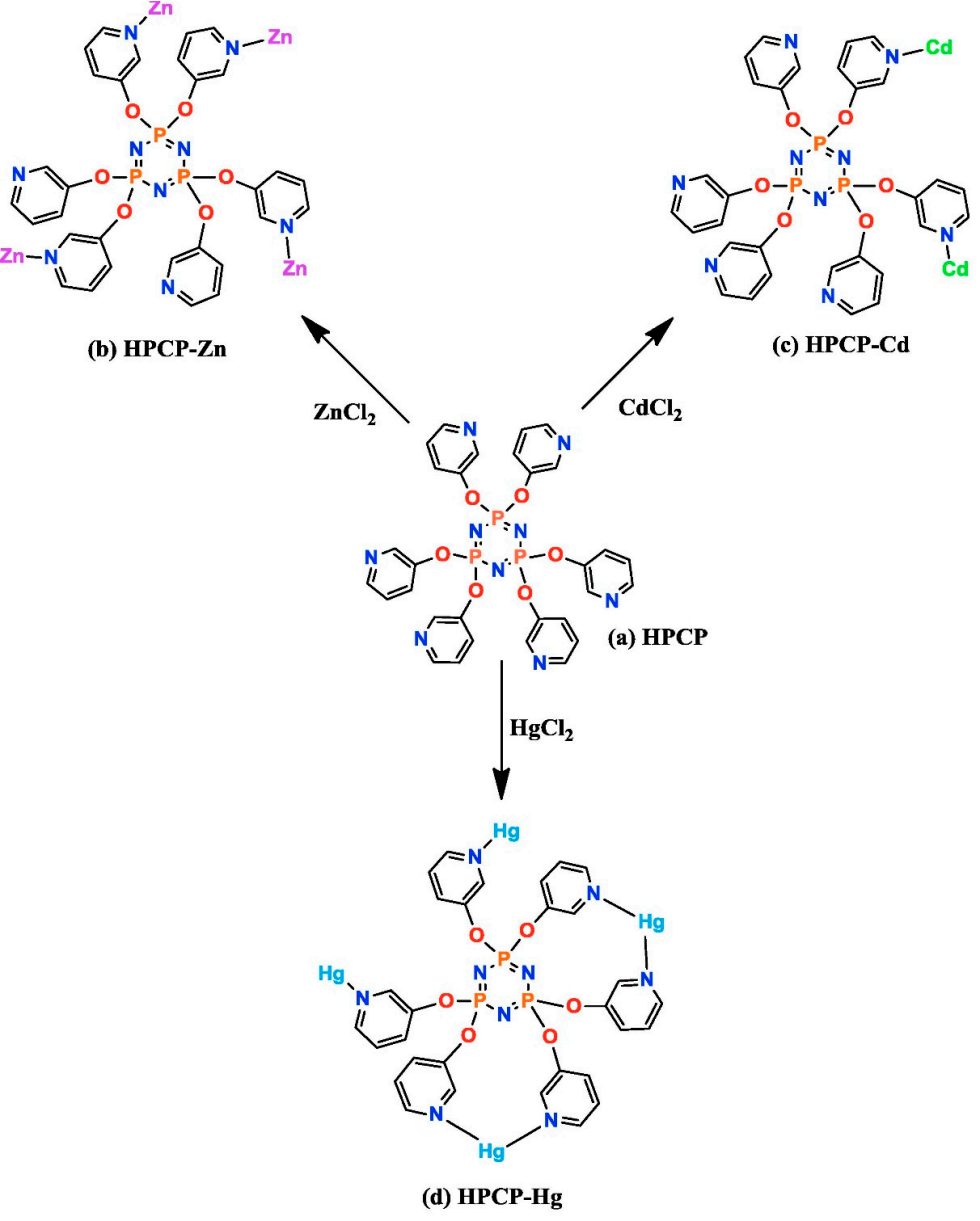
HPCP one, two, and three modalities of coordination. Reproduced with permission from reference [27]. Copyright 2017 Elsevier.

**Figure 17:**
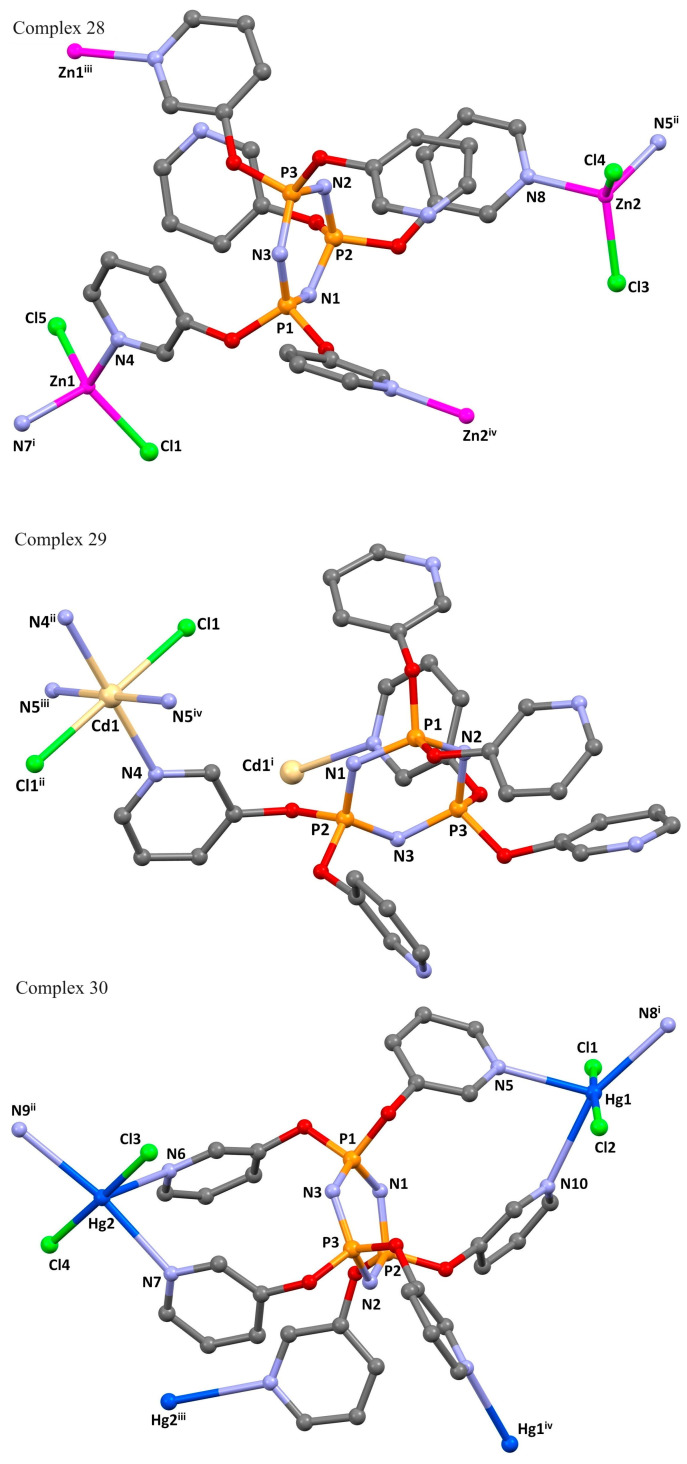
Complexes **28**, **29**, and **30**’s crystal structures [27]. Reproduced with permission from reference [27]. Copyright 2017 Elsevier.

In a different investigation by the same group, Zn, Cd, and Hg complexes of HPCP were synthesized in order to examine the role of the central metal ion in the creation of CPs (Scheme 20). SCXRD was used to identify the structural composition of crystalline substances. The Zn^2+^ (**28**), Cd^2+^ (**29**), and Hg^2+^ (**30**) complexes possess a monoclinic structural structure, and all of the metal atoms belong to the same group (Figure 17). Coordination polymers together with 1D, 2D, and 3D networks may develop as the cyclotriphosphazene ligand self-assembles with the Zn, Cd, and Hg ions. In our investigation, we witnessed the creation of 2D, as well as, 3D CPs. Contrary to the 2D crystal structures of compounds **29** and **30**, compound **28** is curiously organized in a 3D framework made up of two interpenetrating 3D nets.

**Scheme 21:**
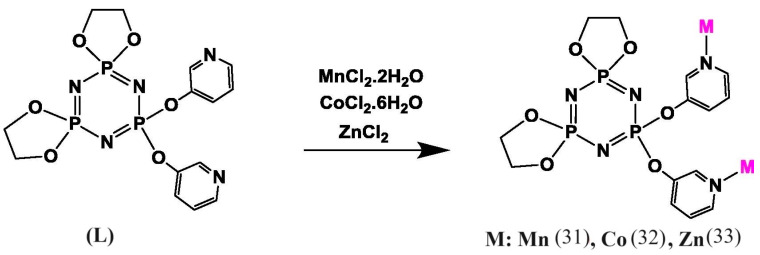
Reaction of L with metal salt. Reproduced with permission from reference [31]. Copyright 2018 Elsevier.

**Figure 18:**
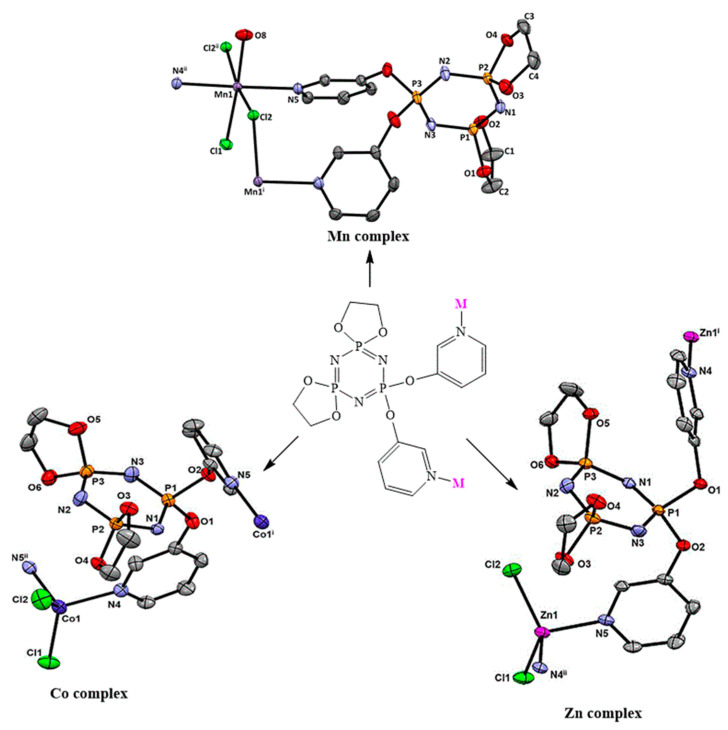
The crystal structures of the Mn, Zn, and Co complexes, as well as the molecular structure of the organic ligand L. Reproduced with permission from reference [31]. Copyright 2018 Elsevier.

The coordinating polymeric compounds, {[Mn(L)(Cl)_2_(H_2_O)]·(CH_3_)_2_CO)}*_n_* (**31**), {[Co(L)(Cl)_2_]}*_n_* (**32**), and {[Zn(L)(Cl)_2_]}*_n_* (**33**), were also reported by the same group [23] and came from the self-assembly processes of the ligand (L) (Scheme 21). By using thermal analyses (TGA), XRD, SCXRD, and elemental analysis, including FTIR, the structural composition of every crystalline molecule was identified. By using the same κ^2^N coordination binding mode to bind the divalent metal ions (Mn^2+^, Co^2+^, and Zn^2+^), XRD demonstrated that all the complexes formed in the monoclinic crystal system were bound to the *P* 2_1_/*c* space group in addition to the ligand (L) to create 1D chain structures (Figure 18). The Co^2+^ and Zn^2+^ions in the complexes have distorted tetrahedral coordination geometry, whereas the core Mn^2+^ion has distorted octahedral coordination geometry. The complexes of Co^2+^ and Zn^2+^ are isostructural.

**Scheme 22:**
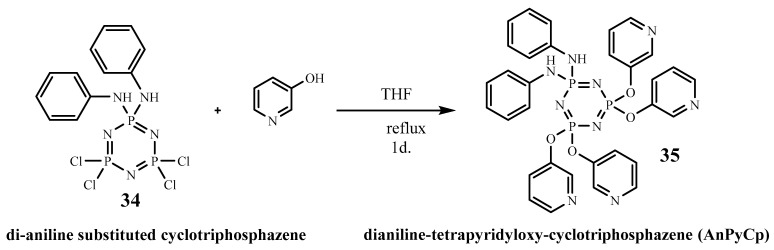
Outlines the AnPyCp ligand’s synthesis process (**35**). Reproduced with permission from reference [105]. Copyright 2019 Elsevier.

In another work, Davarcı et al. [105] successfully synthesized another ligand (AnPyCp) (**35**) by an SN reaction involving 3-hydroxypyridine, and di-aniline substituted HCCP (**34**) was dissolved in dry THF. The synthetic procedure for the AnPyCp ligand is presented in Scheme 22.

**Figure 19:**
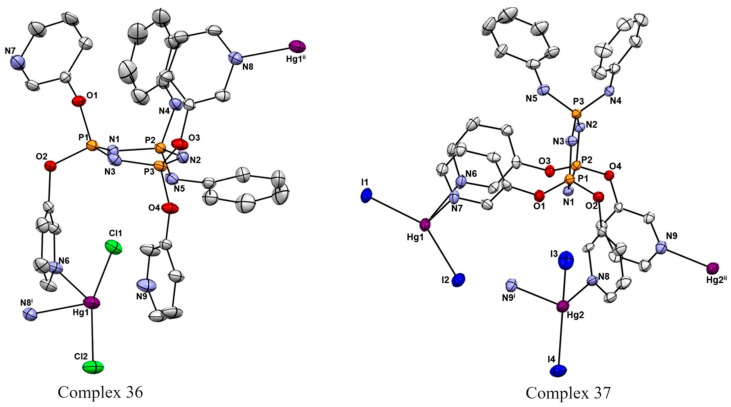
The crystal structures of complexes **36** and **37**. Reproduced with permission from reference [105]. Copyright 2019 Elsevier.

The production of Hg^2+^ complexes with the AnPyCp (**35**) ligand was described in this study [105]. By using FTIR, elemental analysis, and SCXRD, the structures of the isolated crystalline CPs {[Hg(AnPyCp)(Cl)_2_] ·CH_3_CN} *_n_* (**36**) and {[Hg_2_(AnPyCp)(I)_4_] ·CH_3_CN} *_n_* (**37**) were determined. Complexes 1 and 2 form in the triclinic crystal system containing the $P\bar{1}$ space group, according to the investigation of the crystal structure using X-rays. AnPyCp demonstrated κ^2^N and κ^3^N coordination binding modes associated with divalent Hg ions in complexes one and two, resulting in 1D chain structures. In complexes one and two, the shape of the core Hg^2+^ ion is altered (Figure 19).

**Figure 20:**
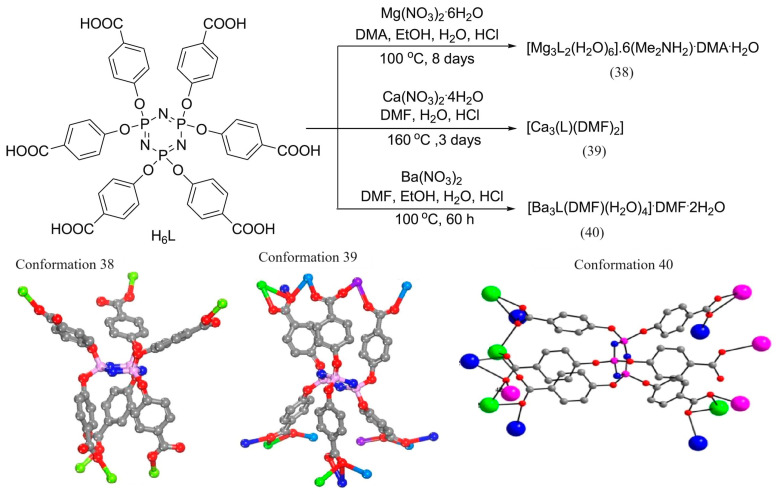
The three alkaline-earth-based coordination polymers’ synthesis process; conformations **38**, **39**, and **40**, as well as the organic ligand H_6_L’s molecular structure, are all discussed. Reproduced with permission from reference [106]. Copyright 2016 Elsevier.

According to Ling et al. [106], three new coordination polymers were produced when H_6_L (Figure 20) was combined with alkaline earth ions of increasing ionic radii (Mg^2+^, Ca^2+^, alongside Ba^2+^) under various solvothermal conditions. SCXRD was used to determine the crystal structures of these three CPs. The Mg compound exhibits a four-connected topological network with the Schläfli symbol of (4^4^·6^2^)_3_(4^9^·6^6^)_2_, which may be explained as a 3D network structure made up of the deprotonated ligand as well as the secondary building block Mg(CO_2_)_4_. The calcium compound is made up of endless 1D “Ca-O” inorganic chains that are joined together from a 3D framework by deprotonated ligands. The 1D “Ba-O” inorganic chains in the barium compound form a 3D framework that is joined by deprotonated organic linkers.

### 2.7. Cyclotriphosphazene Derivatives as Anticorrosive Polymeric Materials

**Figure 21:**
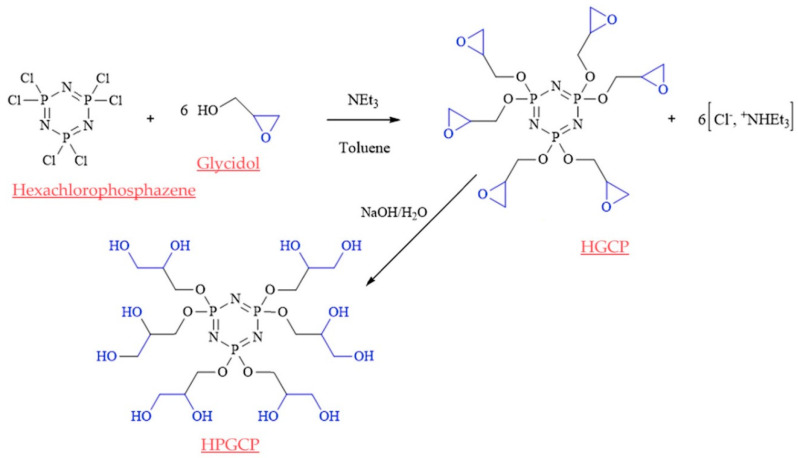
Scheme for the synthesis of hexafunctional prepolymer epoxy (EP) based on cyclotriphosphazene HPGCP. Reproduced with permission from reference [108]. Copyright 2019 Elsevier.

A few papers have portrayed cyclotriphosphazene as an anticorrosive material. In previous papers reported in [6,107,108], a novel hexafunctional epoxy resin (HPGCP) was synthesized based on cyclotriphosphazene according to a reported procedure, which is shown in Figure 21. 

**Figure 22:**
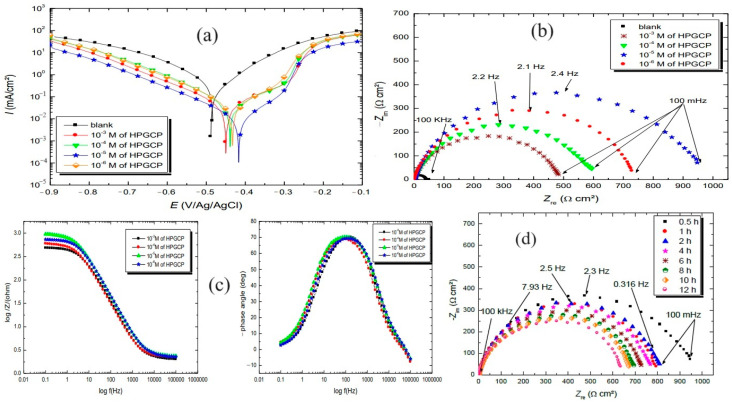
Tafel curves (**a**), Nyquist plots (**b**), and Bode plots (**c**) in 1 M HCl medium with and without HPGCP (**c**), and Nyquist diagrams after different immersion times (**d**) of CS surface without and with 10^−5^ M of HPGCP.

**Figure 23:**
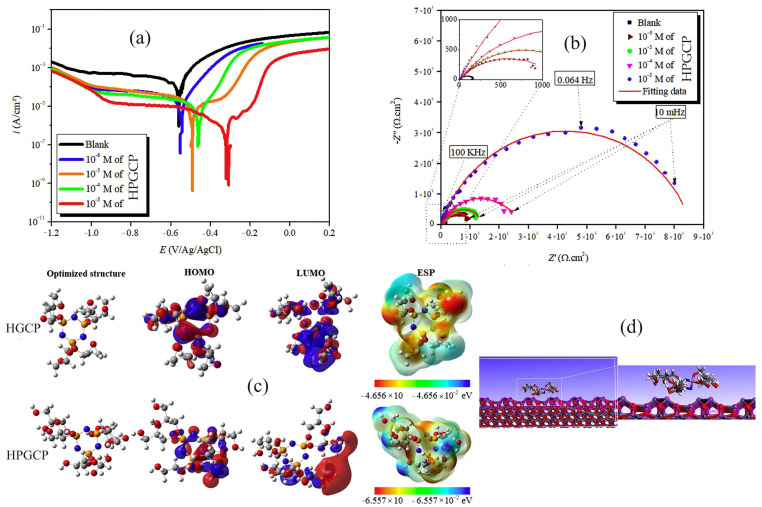
Tafel curves (**a**) and Nyquist plots (**b**) were obtained for CS surface in 3% NaCl corrosive media without and with HPGCP and optimized structures; HOMO, LUMO, and ESP pictures (**c**) and MD results were derived from the adsorption configurations of HPGCP (**d**). Reproduced with permission from reference [108]. Copyright 2019 Elsevier.

HPGCP resin was used as an anticorrosive material for CS and Cu in 1 M HCl and 3% NaCl corrosive media. This inhibitor was studied electrochemically, and the obtained results are presented in Figure 22 and Figure 23. The best corrosion efficiency was 97% at 10^−5^ M in 1 M HCl and 99% at 3% NaCl corrosive media. DFT studies have shown that HPGCP collaborates with the Fe surface (**110**) through communications between receptors, restricting anionic gatherings to the electrostatic precipitator (HOMO) and cationic gatherings as electron acceptors (LUMO). The use of MC showed that HPGCP adsorbs on the metal surface in a horizontal position. Negative *E*_ads_ values show that HPGCP adsorption happens spontaneously.

**Figure 24:**
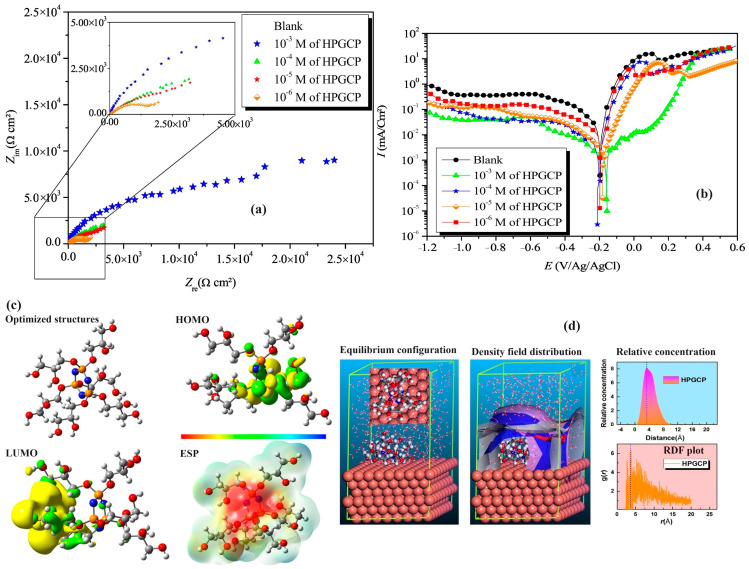
Tafel curves (**a**) and Nyquist plots (**b**) were obtained for copper surface in 3% NaCl corrosive media without and with HPGCP of and optimized structures; HOMO, LUMO, and ESP pictures (**c**) and MD results were derived from the adsorption configurations of HPGCP (**d**). Reproduced with permission from reference [107]. Copyright 2020 Elsevier.

On the other hand, the second HPGCP was investigated for Cu corrosion in corrosive conditions containing 3% NaCl. The anticorrosion properties of HPGCP for Cu corrosion in 3% NaCl were studied by electrochemical and computational techniques, as shown in Figure 24. The results showed that the inhibitor superbly inhibits copper corrosion in 3% NaCl at 10^−3^ M and shows 95% and 96.42% for PDP and EIS, respectively. DFT-based computational investigations show that HPGCP cooperates with the Cu surface through the interaction between adsorption regions, where N molecules of a three-membered ring of cyclotriphosphazene and six π-electrons are shared to vacant d-orbitals of copper. MD exhibits that HPGCP adsorbs on the metal surface using a horizontal alignment. The negative *E*_ads_ values show the chemical interaction of HPGCP with the metal surface through chemisorption processes.

**Figure 25:**
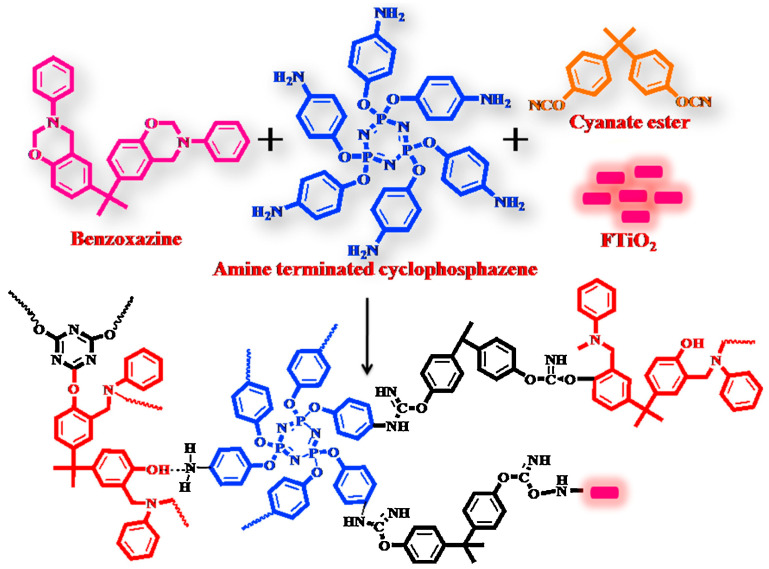
ATCP/FTiO_2_/Bz-CE nanocomposite schematic illustration. Reproduced with permission from reference [109]. Copyright 2016 Elsevier.

In Krishnadevi et al. [109], MS was ready by drenching submersion of amino-terminal cyclotriphosphazene (CP) and actuated titanium dioxide (FTiO_2_) along with benzoxazine nanocomposite cyanate ester (ATCP/FTiO_2_/Bz-CE). The schematic diagram of the arrangement of ATCP/FTiO_2_/Bz-CE nanocomposite is displayed in Figure 25. 

**Figure 26:**
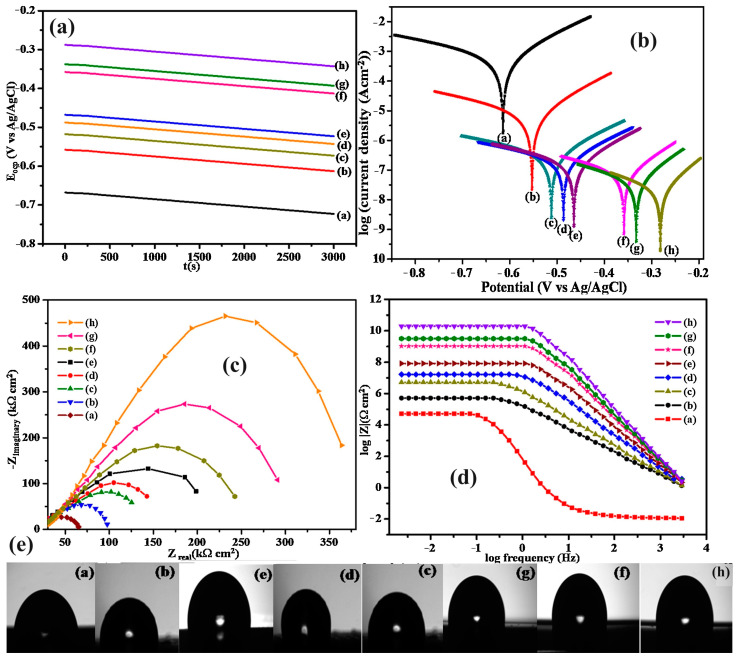
*E*_ocp_ time curves (**a**), Tafel plots (**b**), EIS measurements (**c**), Bode graphs (**d**), and contact angle measurements (**e**) for various coating samples in 3.5% NaCl corrosive media. Reproduced with permission from reference [109]. Copyright 2016 Elsevier.

The anticorrosion abilities of the covering materials were studied utilizing *E*_ocp_ time bends, diagram, EIS estimation including Bode-plots, PDP analysis, and water contact angle measurements in various coverings, testing in a 3.5% NaCl arrangement, as displayed in Figure 26.

The outcomes showed that the useful protection of all MS substrates covered with ATCP/FTiO_2_/Bz-CE nanocomposites (NCs) was over 97%. The outcomes inferred that NCs covered MS were viewed as chemically stable for extensive immersion in destructive conditions.

**Figure 27:**
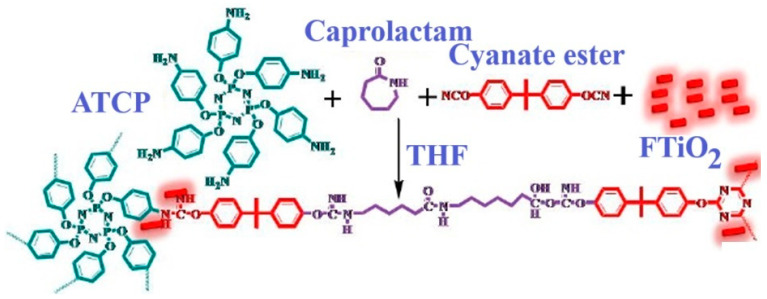
ATCP/FTiO_2_/CE-Cpl composite schematic representation. Reproduced with permission from reference [110]. Copyright 2017 Taylor & Francis.

Krishnadevi et al. [110] examined other nanocomposite materials containing bioactive amine-terminated cyclotriphosphazene (ATCP), functionalized TiO_2_ (FTiO_2_) nanoparticles reinforced caprolactam (Cpl) based cyanate ester (CE) composites (ATCP/FTiO_2_/CE-Cpl) for covering and MS by dip-coating followed by heat treatment. A schematic diagram of the ATCP/FTiO_2_/CE-Cpl composite development is displayed in Figure 27. 

**Figure 28:**
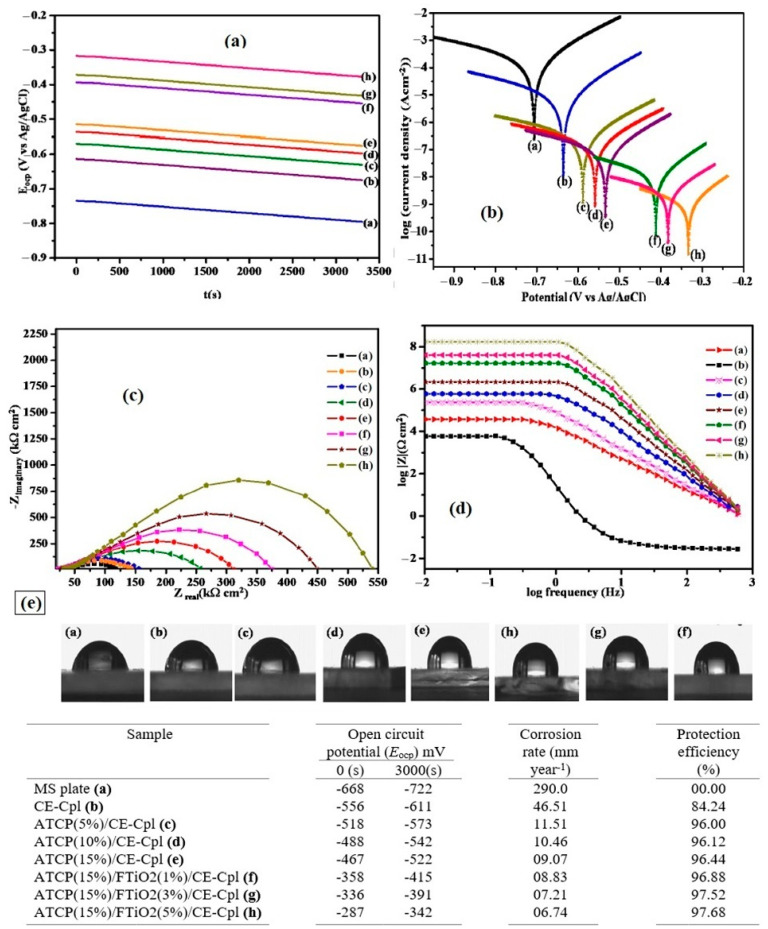
*E*_ocp_ time curves (**a**), Tafel plots (**b**), EIS measurements (**c**), Bode graphs (**d**), and contact angle measurements (**e**) for various covering samples in 3.5 wt% NaCl corrosive media. Reproduced with permission from reference [110]. Copyright 2017 Taylor & Francis.

ATCP/FTiO_2_/CE-Cpl composites were applied on the surface of MS plates; their anticorrosive properties were analyzed utilizing *E_ocp_* time bend, EIS estimation including Bode plots, PDP analysis, and water contact angle measurements in a 3.5% NaCl arrangement, as displayed in Figure 28. The outcomes showed that the MS substrates were effectively covered with ATCP/FTiO_2_/CE-Cpl nanocomposites with a 96% protection degree. ATCP/FTiO_2_/CE-Cpl nanocomposites have further developed corrosion resistance abilities because of the presence of FTiO_2_ and ATCP nanoparticles.

**Figure 29:**
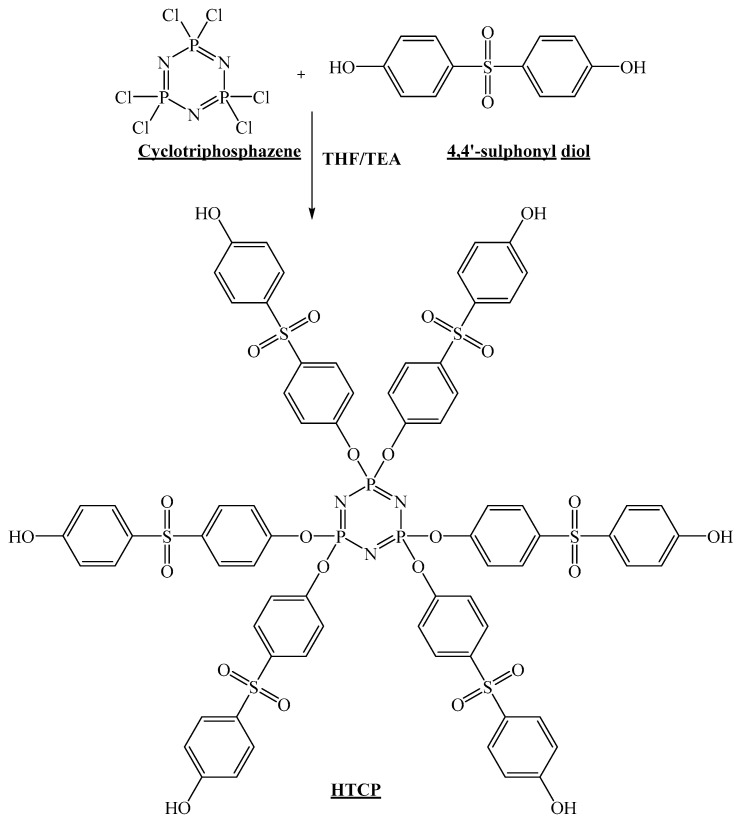
Creating the HTCP.

**Figure 30:**
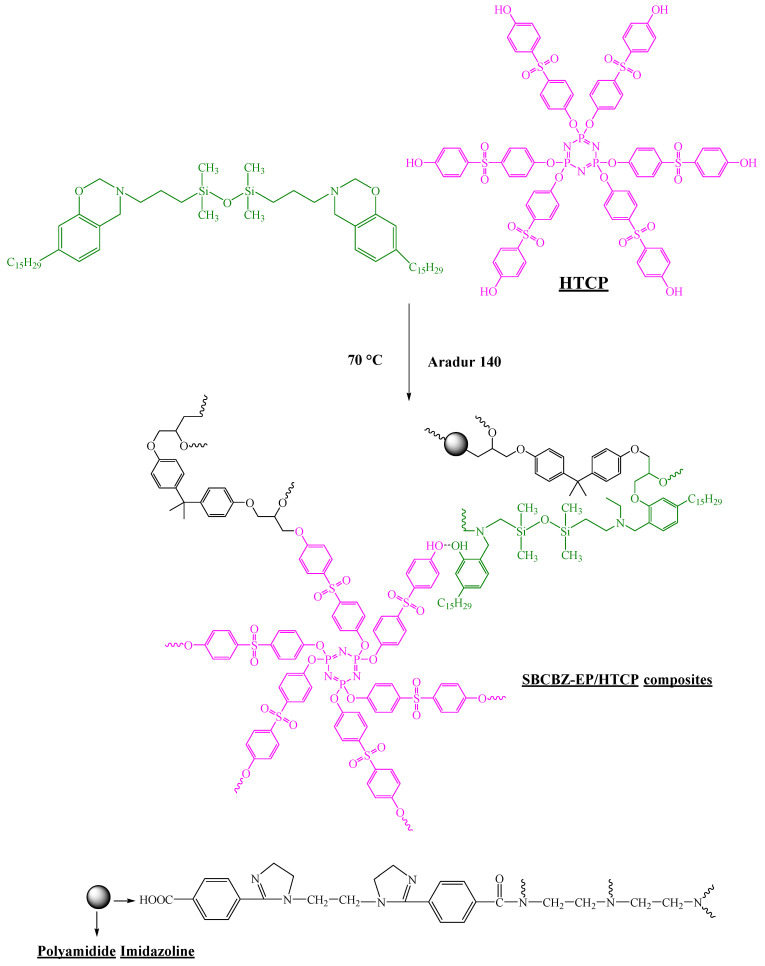
Creation of SBCBz-EP hybrid composites reinforced with HTCP.

When compared to standard benzoxazines, Selvaraj et al. [111] produced and analyzed the SBCBz-EP hybrid matrix reinforced using HTCP composites capable of curing at very low temperatures. Figure 29 shows the schematic diagram for the creation of HTCP, and Figure 30 shows the creation of HTCP-reinforced SBCBz-EP hybrid composites. Numerous weight percentages (one, three, and five wt%) of HTCP were used to strengthen the matrix SBCBz-EP, and various analytical methods were used to describe the resultant hybrid composites. Data from electrochemical investigations reveal that the addition of nitrogen, phosphorous, and aromatic structure considerably enhanced the corrosion-resistant capabilities of hybrid composites (SBCBZ-EP/HTCP). Additionally, the findings of electrochemical experiments indicated that (SBCBZ-EP/HTCP) composites might be employed as polymeric coatings for high-performance industrial applications due to their low cure temperature (70 °C).

#### Mechanism of Action of Cyclotriphosphazene-Based Corrosion Inhibitors

A few techniques have been proposed to repress metal corrosion of heterocyclic inhibitors. It is by and large acknowledged that the initial step of the inhibitory impact depends on the adsorption of the metal surface in the acidic medium [112,113,114]. The adsorption procedure of inhibitors is affected by the idea of the metal surface, the compound design of the heterocyclic inhibitor, the worth of the molecular dispersion, the kind of corrosive electrolyte, and the idea of the cooperation between the heterocyclic particles and the metal surface [115,116]. In most restraint studies, the arrangement of the electron receptor on the d-orbital of the inhibitor was seen on the outer layer of the metal [91]. Nitrogen-based compounds are successful in repressing corrosion in fluid arrangements [117,118]. The presence of a solitary electron nitrogen particle makes the energy change during the expansion of delocalized nitrogen molecules that balance out the compound. It is likewise realized that heterocyclic nitrogen mixtures can be adsorbed by electrostatic collaboration between positive nitrogen ions and a negative metal [119,120]. It is additionally realized that the adsorption of inhibitors can be impacted by the idea of anions and acids [121]. They are described by solid adsorption on the metal surface, prompting adverse consequences and advancing the adsorption of cationic inhibitors [122,123].

**Figure 31:**
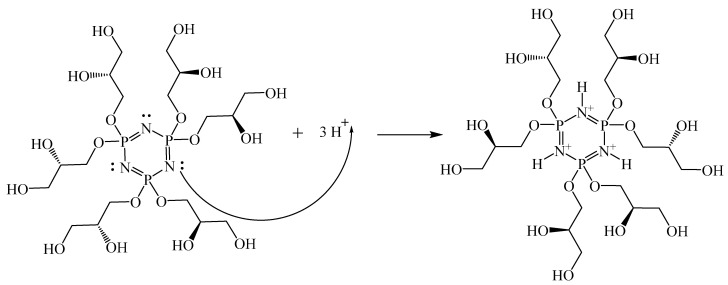
Molecular form of HPGCP in an acid medium.

The function of HPGCP/HCl can be described as follows. In aqueous acidic compounds, HPGCP is present in medium or cationic molecules, as shown in Figure 31.

**Figure 32:**
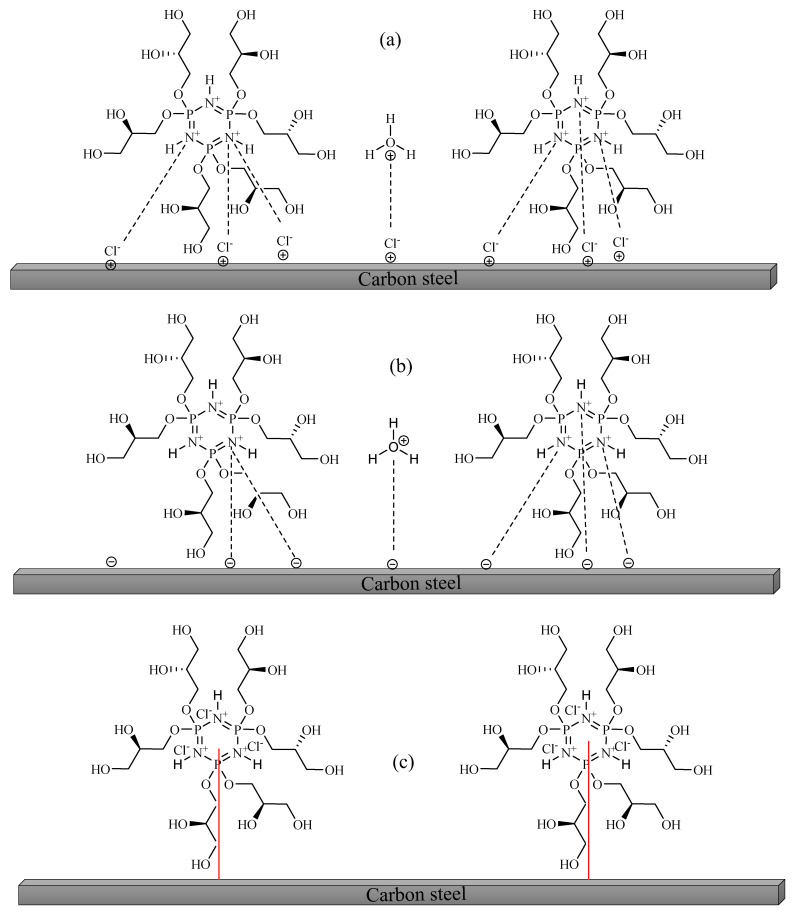
Schematic portrayal of HPGCP adsorption on carbon steel in 1M HCl arrangement: (**a**) CS surface with a positive charge, (**b**) CS surface with a negative charge, and (**c**) CS surface at a potential of zero charges.

Consequently, the adsorption of HPGCP as a halfway particle on the metal surface can happen because of the immediate exchange of H_2_O particles from the metal surface and the trading of electrons between nitrogen molecules and the metal surface. These heterocyclic nitrogen (N) compounds are additionally consumed by the electrostatic communication between the emphatically charged N molecule and the contrarily charged metal surface. A schematic portrayal of the adsorption of HPGCP on carbon steel (CS) in a 1 M HCl arrangement is displayed in Figure 32. Whenever a carbon steel structure is in an HCl arrangement containing HPGCP, three kinds of particles are adsorbed on a superficial level. At the point when the metal surface is all around charged (Figure 32a), comparable with the zero potential (PZP), chloride particles are typically adsorbed on the metal surface, which accordingly draws in cationic substances from HPGCP and protonated water atoms. For this situation, a tight three-layer metal layer is framed, which keeps metal particles from entering the arrangement. Subsequently, working on the nature of the metal surface will expand the adsorption of HPGCP and decline the HPGCP in the arrangement. At the point when the metal surface is troublesome (Figure 32b), the cationic substance of protonated water and PHGC particles is adsorbed straightforwardly on the metal surface. As the negative surface area of the metal builds, the adsorption of HPGCP increments and the grouping of the substance diminishes. 

When the metal drops to the surface charge potential of zero, the ionic ions on the surface do not adsorb any ions (no cations or anions). However, some HPGCP molecules will adsorb due to the flat p π-orbitals of the metal surface (which has the π-orbitals of free radicals) (Figure 32c).

**Figure 33:**
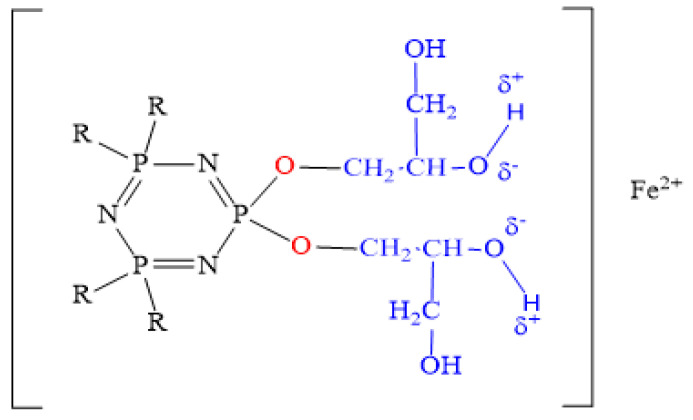
Application of polarization in carbon steel surface design.

HPGCP adsorption on metal surfaces in sodium chloride (NaCl) can be accomplished straight by the connection of gas receptors and the vacant d-orbital of steel surface particles or by the communication of organo-nitrogen mixtures and adsorbed gatherings. Following the beginning of the consumption response, the metal is encircled by two oxygen molecules, as displayed in Figure 33.

**Figure 34:**
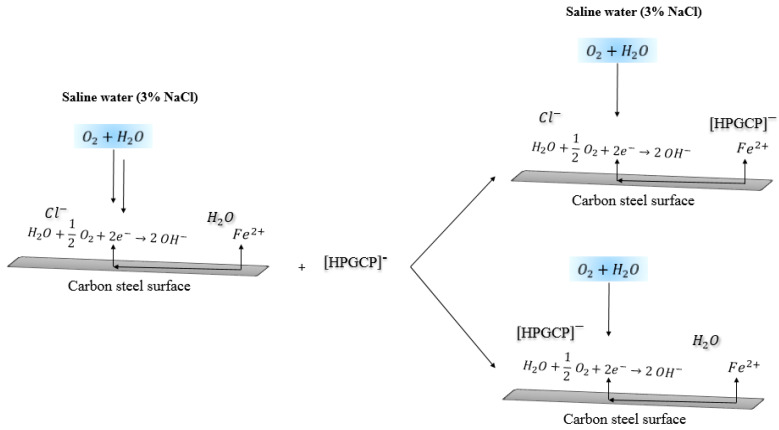
Schematic representation of competitive adsorption of HPGCP in sodium chloride.

The following description can be assumed: HPGCP interacts with the separation reaction during adsorption on the metal surface. The inhibitor competes with water molecules and Cl^−^ ion for the surface area of the water-coated anode, as shown in Figure 34.

## 3. Research Gaps Identified

As such, there is a need for an in-depth insight into the mechanism of adsorption of cyclotriphosphazene derivatives compounds on metal surfaces. Despite the potential shown by coordination compounds as anticorrosive polymeric materials, their applications as corrosion inhibitors and coatings in aqueous electrolytes remain very minimal. 

Therefore, the design and synthesis of varieties of coordination compounds based on cyclophosphazene derivatives as anticorrosive polymeric materials suitable for several alloys in diverse corrosive environments is favorably recommended.

Protection efficacies of coordination compounds based on cyclophosphazene derivatives could further be enhanced by varying the primary ligands moiety during design; hence, machine learning and quantitative structure–activity relationship models could be employed.

## 4. Conclusions

In conclusion, this review paper has delved into the dynamic landscape of cyclophosphazenes, emphasizing significant progress made in their preparation, elucidation of properties, and diverse applications. The following key conclusions can be highlighted:

- The evolution of synthetic methodologies underscores the versatility of cyclophosphazenes, showcasing advancements in tailored design and enhanced control over their structural features;
- The comprehensive examination of their distinctive properties, including thermal stability attributes, further accentuates their appeal in various applications;
- The exploration of cyclophosphazenes in different domains, from materials science to medicinal applications, highlights the expanding scope and potential impact of these compounds;
- As evidenced by the breadth of research discussed, cyclophosphazenes continue to captivate the scientific community, offering promising avenues for innovation and practical utilization;
- Focus on the most recent developments in coordination chemistry centered around cyclotriphosphazene derivatives;
- Substituted cyclotriphosphazene derivatives serve as advantageous synthons in coordination chemistry, highlighting their importance in this field;
- The general information provided on cyclotriphosphazene derivatives as anticorrosive polymeric materials;
- Overview of trends in the synthesis of cyclotriphosphazene derivatives and recent progress in this area;
- Discussion of the anticorrosion performances of cyclotriphosphazene derivatives, emphasizing their potential as protective materials;
- Mention of heteroatoms such as O, N, and P, including the aromatic part, contributing to the availability of cyclotriphosphazene derivatives as effective protective materials;
- Recognition of polymeric materials derived from cyclotriphosphazenes for their superior surface adsorption and corrosion prevention properties;
- The assertion that polymeric materials based on cyclotriphosphazenes exhibit higher anticorrosive properties compared to other organic corrosion inhibitors;
- Explanation of how the excellent physical adsorption capacities of polymeric materials derived from cyclotriphosphazenes protect metals from corrosive environments.

## 5. Future Prospects

The following were discovered to be the primary cyclotriphosphazene derivatives for anticorrosive polymeric materials in the future:

The effectiveness of cyclotriphosphazene encourages future corrosion inhibitors along with anticorrosive coating applications to have excellent anticorrosive properties. 

The development of superior corrosion inhibitors along with anticorrosive coating applications is credited to the chemical transformation of cyclotriphosphazene with a variety of aromatic compounds as well as functional groups.

The cyclotriphosphazene-based chemicals would enable further investigation into the corrosion of steel, copper, and aluminum. This is because anticorrosive coating applications and cyclotriphosphazene-based corrosion inhibitors have excellent chelating performance.

Future studies will modify the cyclotriphosphazene rings with surfactants, ionic liquids, epoxy resins, carbon dots, graphene oxide, and nanocarriers, which would be important in the study of corrosion inhibition.

## Data Availability

Not applicable.
